# Weak neuronal glycolysis sustains cognition and organismal fitness

**DOI:** 10.1038/s42255-024-01049-0

**Published:** 2024-05-24

**Authors:** Daniel Jimenez-Blasco, Jesús Agulla, Rebeca Lapresa, Marina Garcia-Macia, Veronica Bobo-Jimenez, Dario Garcia-Rodriguez, Israel Manjarres-Raza, Emilio Fernandez, Yannick Jeanson, Spiro Khoury, Jean-Charles Portais, Daniel Padro, Pedro Ramos-Cabrer, Peter Carmeliet, Angeles Almeida, Juan P. Bolaños

**Affiliations:** 1 Institute of Functional Biology and Genomics, Universidad de Salamanca, CSIC, Salamanca, Spain; 2 Institute of Biomedical Research of Salamanca (IBSAL), Hospital Universitario de Salamanca, Universidad de Salamanca, CSIC, Salamanca, Spain; 3 Centro de Investigación Biomédica en Red de Fragilidad y Envejecimiento Saludable, Madrid, Spain; 4 RESTORE, University of Toulouse, Inserm U1031, CNRS 5070, UPS, EFS, Toulouse, France; 5 MetaboHUB-MetaToul, National Infrastructure of Metabolomics and Fluxomics, Toulouse, France; 6 Toulouse Biotechnology Institute, INSA de Toulouse INSA/CNRS 5504, UMR INSA/INRA 792, Toulouse, France; 7 CIC biomaGUNE, Basque Research and Technology Alliance, Donostia-San Sebastián, Spain; 8 Ikerbasque, Basque Foundation for Science, Bilbao, Spain; 9 Laboratory of Angiogenesis and Vascular Metabolism, Department of Oncology and Leuven Cancer Institute (LKI), KU Leuven, VIB Center for Cancer Biology, VIB, Leuven, Belgium; 10 Center for Biotechnology, Khalifa University of Science and Technology, Abu Dhabi, United Arab Emirates

**Keywords:** Molecular neuroscience, Neurochemistry

## Abstract

The energy cost of neuronal activity is mainly sustained by glucose^1,2^. However, in an apparent paradox, neurons modestly metabolize glucose through glycolysis^3–6^, a circumstance that can be accounted for by the constant degradation of 6-phosphofructo-2-kinase–fructose-2,6-bisphosphatase-3 (PFKFB3)^3,7,8^, a key glycolysis-promoting enzyme. To evaluate the in vivo physiological importance of this hypoglycolytic metabolism, here we genetically engineered mice with their neurons transformed into active glycolytic cells through *Pfkfb3* expression. In vivo molecular, biochemical and metabolic flux analyses of these neurons revealed an accumulation of anomalous mitochondria, complex I disassembly, bioenergetic deficiency and mitochondrial redox stress. Notably, glycolysis-mediated nicotinamide adenine dinucleotide (NAD^+^) reduction impaired sirtuin-dependent autophagy. Furthermore, these mice displayed cognitive decline and a metabolic syndrome that was mimicked by confining *Pfkfb3* expression to hypothalamic neurons. Neuron-specific genetic ablation of mitochondrial redox stress or brain NAD^+^ restoration corrected these behavioural alterations. Thus, the weak glycolytic nature of neurons is required to sustain higher-order organismal functions.

## Abstract (web summary)

Jiménez-Blasco et al. show that neurons exhibit moderately low glycolytic rates despite their activity being mainly supported by glucose to preserve redox balance.

## Main

**Fig. 1:**
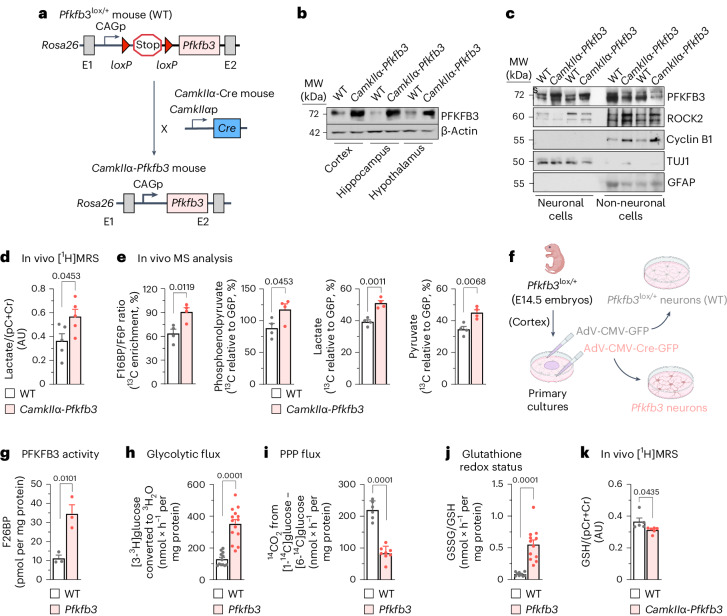
In vivo neuron-specific *Pfkfb3* expression activates glycolysis and inhibits the PPP, causing redox stress. **a**, Strategy used to generate neuron-specific *Pfkfb3*-expressing (*CamkIIα-Pfkfb3*) mice. p, promoter. **b**, Western blot for PFKFB3 protein in different areas of the *CamkIIα-Pfkfb3* mouse brain. β-Actin was used as a loading control (Extended Data Fig. 1b). MW, molecular weight; WT, wild type. **c**, Western blot for PFKFB3 protein in neurons and non-neuronal cells immunomagnetically isolated from *CamkIIα-Pfkfb3* mice. GFAP and TUJ1 were used as astrocyte- or neuron-enrichment and loading controls (Extended Data Fig. 1d). **d**, In vivo [^1^H]MRS analysis of the lactate/(pCr+Cr, phosphocreatine+creatine) ratio in the brain of *CamkIIα-Pfkfb3* mice. Data are mean ± s.e.m. *P* value is indicated (*n* = 5 mice per genotype; unpaired Student’s *t*-test, two sided) (Extended Data Fig. 1e). AU, arbitrary units. **e**, In vivo ^13^C-enrichment MS analysis of the F16BP/F6P ratio and the indicated glycolytic intermediates in the brain of *CamkIIα-Pfkfb3* mice. Data are mean ± s.e.m. *P* value is indicated (*n* = 4 mice per genotype; unpaired Student’s *t*-test, two sided) (Extended Data Fig. 1f). **f**, Adenoviral transduction strategy used to express *Pfkfb3* in brain cortical neurons in primary culture from *Pfkfb3*^lox/+^ mice. Created with https://www.biorender.com. E, embryonic day. **g**–**j**, F26BP concentration (**g**), glycolytic flux as measured by the rate of [3-^3^H]glucose conversion into ^3^H_2_O (**h**), PPP flux as measured by the difference in ^14^CO_2_ production from [1-^14^C]glucose and [6-^14^C]glucose (**i**) and glutathione redox status as measured by the ratio of GSSG versus GSH (**j**) in primary neurons. Data are mean ± s.e.m. *P* value is indicated; *n* = 3 (**g**), *n* = 12 (**h**, WT), *n* = 14 (**h**, *Pfkfb3*), *n* = 6 (**i**, WT), *n* = 8 (**i**, *Pfkfb3*), *n* = 9 (**j**, WT) and *n* = 12 (**j**, *Pfkfb3*) biologically independent cell culture preparations; unpaired Student’s *t*-test, two sided (Extended Data Fig. 1h,i). **k**, In vivo [^1^H]MRS analysis of the GSH/(pCr+Cr) ratio in the brain of *CamkIIα-Pfkfb3* mice. Data are mean ± s.e.m. *P* value is indicated; *n* = 5 (WT) and *n* = 6 (*CamkIIα-Pfkfb3*) mice; unpaired Student’s *t*-test, two sided. Source data

**Extended Data Fig. 1:**
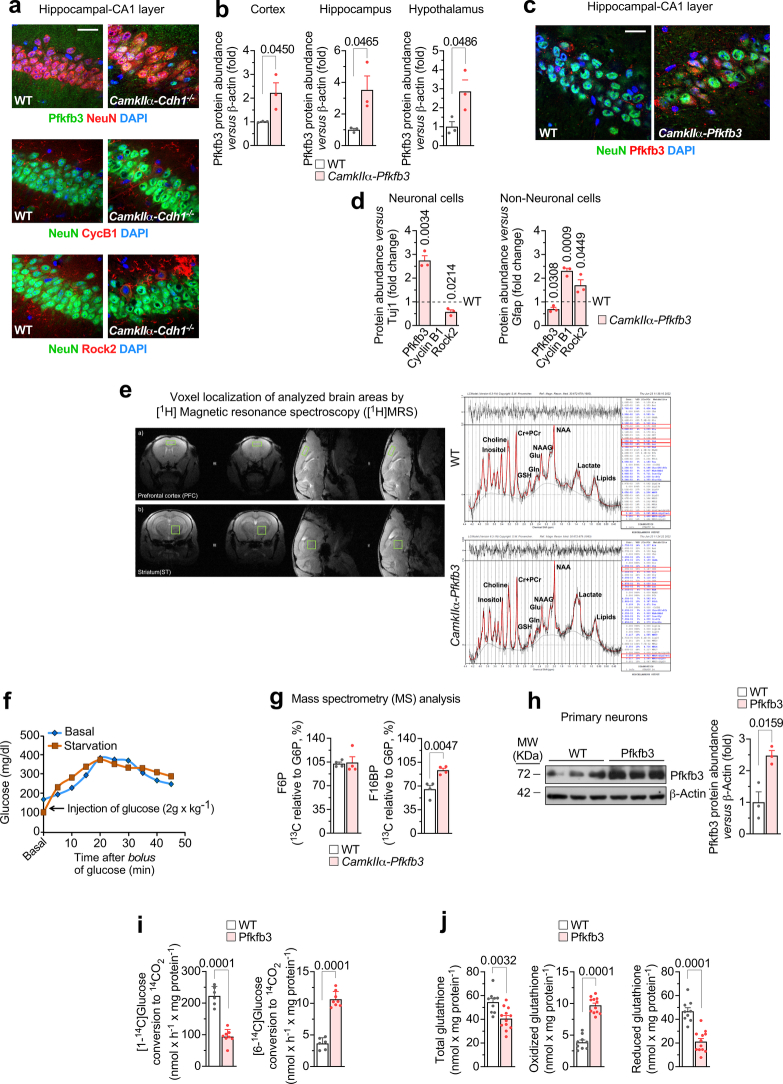
*In vivo* neuron-specific *Pfkfb3* expression activates glycolysis and inhibits PPP causing redox stress. **(a)** Immunocytochemical confocal images in neuron-specific Cdh1 knockout mice (*CamkIIα-Cdh1*^−*/*−^). NeuN and DAPI identify neurons and nuclei, respectively. Bar=20 µm (n = 3 mice per genotype). **(b)** Western blot quantifications for Fig. 1b. Data are mean ± S.E.M. *P* value is indicated (n = 3 mice per genotype; Unpaired Student’s *t*-test, two-sided). (Uncropped western blot replicas are shown as Source Data file). **(c)** Immunocytochemical confocal images in *CamkIIα-Pfkfb3* mice. Bar=20 µm. (n = 3 mice per genotype). **(d)** Western blot quantifications for Fig. 1c. Data are mean ± S.E.M. *P* value is indicated (n = 3 mice per genotype; Unpaired Student’s *t*-test, two-sided). (Uncropped western blot replicas are shown as Source Data file). **(e)** Voxel localization of [^1^H]MRS analyzed brain areas (left) and representative ^1^H spectrum (right). Related to Fig. 1d,l. **(f)** Preliminary experiment for the ^13^C-glucose-mass spectrometry (MS) analysis. WT fed or fasted (6 h) mice were injected (i.p.) with a *bolus* of glucose (2 g/kg), and blood glucose was determined to estimate the time at which glucose levels reached the steady-state concentration (~20 min). Data are average values from 2 technical replicas in 2 mice. Related to Fig. 1e. **(g)** *In vivo* F6P and F16BP concentrations analyzed by MS in the mice brain extracts. Data are mean ± S.E.M. *P* value is indicated (n = 4 mice per genotype; Unpaired Student’s *t*-test, two-sided). Related to Fig. 1e. **(h)** Western blot against Pfkfb3 protein and quantification. ß-Actin, loading control. Data are mean ± S.E.M. *P* value is indicated (n = 3 mice per genotype; Unpaired Student’s *t*-test, two-sided). (Uncropped western blot replicas are shown as Source Data file). **(i)** ^14^CO_2_ production from [1-^14^C]glucose (left) and [6-^14^C]glucose (right) in primary neurons. Data are mean ± S.E.M. *P* value is indicated; n = 6 (WT) or 8 (Pfkfb3) biologically independent cell culture preparations; Unpaired Student’s *t*-test, two-sided). Related to Fig. 1j. **(j)** Total (left), oxidized (middle) and reduced (right) glutathione concentrations in primary neurons. Data are mean ± S.E.M. *P* value is indicated; n = 9 (WT) or 12 (Pfkfb3) biologically independent cell culture preparations; Unpaired Student’s *t*-test, two-sided). Related to Fig. 1k. Source data

The proteolytic destabilization of PFKFB3 in neurons takes place upon polyubiquitination by the E3 ubiquitin ligase APC/C (anaphase-promoting complex, also known as the cyclosome)^3^. APC/C can be activated by either of the cofactors CDH1 (cell division cycle 20 homolog 1) or CDC20 (cell division cycle 20)^7^. However, CDC20 is not present in differentiated neurons^8^; hence, CDH1 is the only cofactor responsible for the observed high APC/C activity that leads to PFKFB3 protein destabilization and neuronal hypoglycolysis^3^. Stabilization of endogenous neuronal PFKFB3 takes place in several disease conditions^9,10^ and during development^6^. In addition, aberrant hyperglycolysis takes place in Alzheimer’s disease neurons^11^. However, the in vivo physiological importance of weak adult neuronal glycolysis remains unknown, a limitation that has led to controversies^1^, thus hindering a better knowledge of brain function in health and disease. To address this issue, we aimed to generate a genetic mouse model able to boost glycolysis in neurons by means of *Pfkfb3* expression during adulthood. First, conditional *Cdh1*-knockout (*Cdh1*^lox/lox^) mice^12^ were mated with mice expressing Cre recombinase under the control of the neuron-specific *Camk2a* (also known as *CamkIIα*) promoter, which is widely expressed across the brain, mainly in the hippocampus, neocortex, striatum and amygdala as from the third postnatal week^13^. As expected, *CamkIIα-Cdh1*^−/−^ progeny showed PFKFB3 protein stabilization in neurons of the adult brain (Extended Data Fig. 1a). However, cyclin B1 and Rho-associated coiled-coil-containing protein kinase 2 (ROCK2), that is, two proteins known to be APC/C–CDH1 substrates that cause neurotoxicity^8^ and cognitive decline^14^, were also found to be stabilized (Extended Data Fig. 1a). These data did not persuade us that inhibition of APC/C–CDH1 activity would be a suitable strategy to specifically assess the in vivo impact of active glycolysis in neurons. To overcome this issue, we next generated, by homologous recombination in the *Rosa26* locus, a transgenic mouse harbouring the full-length cDNA for *Pfkfb3* (Fig. 1a). A floxed (*loxP*-flanked) transcriptional stop cassette was incorporated between *Pfkfb3* cDNA and the CAG promoter (*Pfkfb3*^lox/+^) to eventually obtain tissue- and time-specific expression of *Pfkfb3* in vivo (Fig. 1a). To ascertain the efficacy of this strategy, *Pfkfb3*^lox/+^ mice were mated with *CamkIIα-cre* mice to generate *CamkIIα-Pfkfb3* (Fig. 1a) mice. *Pfkfb3*^lox/+^ littermates were used as controls (wild type; WT) (Fig. 1a). *CamkIIα-Pfkfb3* mouse brains showed enhanced PFKFB3 protein levels, as judged by western blotting of all areas analysed such as the cortex, hippocampus and hypothalamus (Fig. 1b and Extended Data Fig. 1b) as well as by hippocampal immunocytochemistry (Extended Data Fig. 1c).

In contrast to neurons, APC/C–CDH1 activity is low in astrocytes and accounts for their naturally higher PFKFB3 abundance and glycolytic phenotype^3^. To ascertain whether the increase in PFKFB3 protein in the brain of *CamkIIα-Pfkfb3* mice is neuron specific, neurons and astrocytes were immunomagnetically purified, and PFKFB3 protein levels were analysed in both cell types by western blotting. As shown in Fig. 1c (Extended Data Fig. 1d), the abundance of PFKFB3 protein in *CamkIIα-Pfkfb3* neurons is similar to that found in wild-type astrocytes. This indicates that the abundance of PFKFB3 protein in neurons of the *CamkIIα-Pfkfb3* mouse model is moderately high enough to reach levels comparable with those physiologically found in neighbouring astrocytes. Moreover, sustained *Pfkfb3* expression does not result in overwhelming of APC/C–CDH1 ubiquitin ligase or proteasomal activities, as the protein levels of ROCK2 and cyclin B1 (APC/C–CDH1 substrates^8,14^) do not increase in neurons isolated from *CamkIIα-Pfkfb3* mice (Fig. 1c and Extended Data Fig. 1d). To assess whether PFKFB3 protein stabilization in *CamkIIα-Pfkfb3* neurons is functional, we used two strategies. First, in vivo ^1^H magnetic resonance spectroscopy ([^1^H]MRS) analysis was performed in the *CamkIIα-Pfkfb3* mouse brain (Extended Data Fig. 1e), which revealed an enhancement in the concentration of lactate (Fig. 1d), suggesting increased glycolysis. Second, *CamkIIα-Pfkfb3* mice were intraperitoneally injected with [U-^13^C]glucose, and ^13^C-labelled glycolytic intermediates were assessed in brain extracts by mass spectrometry (MS). As shown in Fig. 1e (Extended Data Fig. 1f,g), the ratio of fructose 1,6-bisphosphate (F16BP) versus fructose 6-phosphate (F6P) (F16BP/F6P), that is, the product and substrate of 6-phosphofructo-1-kinase (PFK1), respectively, was increased, indicating PFK1 activation in the brain of the *CamkIIα-Pfkfb3* mice. Moreover, ^13^C mass spectrometry analysis also revealed that levels of the downstream glycolytic intermediaries phosphoenolpyruvate, pyruvate and lactate were also enhanced in *CamkIIα-Pfkfb3* mice (Fig. 1e), indicating an efficient, otherwise moderate increase in neuronal PFKFB3 protein levels that leads to PFK1 activation and enhanced glycolytic flux in vivo.

To validate glycolytic flux activation in neurons, these cells were cultured from *Pfkfb3*^lox/+^ mouse embryos and, once differentiated, transduced with adenovirus expressing Cre recombinase under the cytomegalovirus (CMV) promoter (adeno-associated virus (AAV)-CMV-Cre-GFP) to generate *Pfkfb3*-expressing (*Pfkfb3*) neurons. *Pfkfb3*^lox/+^ neurons transduced with the AAV lacking Cre recombinase (AAV-CMV-GFP) were used as controls (wild type) (Fig. 1f). As shown in Extended Data Fig. 1h, levels of PFKFB3 protein increased, and its functional activity was confirmed as judged by enhancement in the PFKFB3 product fructose 2,6-bisphosphate (F26BP) in *Pfkfb3* neurons (Fig. 1g). These cells had higher glycolytic flux, as analysed by the rate of [3-^3^H]glucose incorporation into ^3^H_2_O, a bona fide index of glycolysis^3^ (Fig. 1h). As glycolysis and the pentose phosphate pathway (PPP) are inversely correlated in neurons^15^, we determined the PPP flux in *Pfkfb3* neurons by estimating the difference in ^14^CO_2_ production from [1-^14^C]glucose (decarboxylated during the PPP and the tricarboxylic acid (TCA) cycle) and [6-^14^C]glucose (decarboxylated during the TCA cycle^16,17^). As shown in Fig. 1i (Extended Data Fig. 1i), PPP flux decreased in *Pfkfb3* neurons. By producing nicotinamide adenine dinucleotide phosphate (NADPH(H^+^)), the PPP sustains the regeneration of reduced glutathione (GSH) from its oxidized form (GSSG)^17^. Accordingly, GSH (reduced and total forms) levels decreased and GSSG levels increased in *Pfkfb3* neurons, resulting in enhanced glutathione oxidation (increased GSSG/GSH ratio) (Fig. 1j and Extended Data Fig. 1j). These results were confirmed in vivo by brain [^1^H]MRS analysis in *CamkIIα-Pfkfb3* mice, as judged by the decreased GSH signal (Fig. 1k), despite the weaker magnitude that reflects mixed cell types. These data agree with previous observations indicating that the PPP is an advantageous metabolic pathway for neurons^17–19^.

**Fig. 2:**
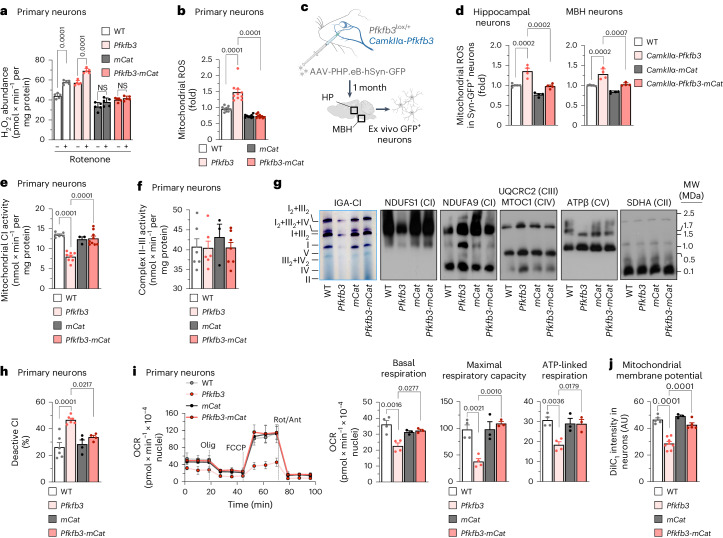
Neuron-specific *Pfkfb3* expression impairs mitochondrial bioenergetics via enhanced mitochondrial ROS. **a**, H_2_O_2_ production. Data are mean ± s.e.m. *P* values are indicated; *n* = 5 biologically independent cell culture preparations; two-way ANOVA followed by Tukey’s test. NS, not significant. **b**, Mitochondrial ROS production. Data are mean ± s.e.m. *P* values are indicated; *n* = 10 (WT, *Pfkfb3*) or *n* = 9 (*mCat*, *Pfkfb3*-*mCat*) biologically independent cell culture preparations; one-way ANOVA followed by Tukey’s test. **c**, In vivo AAV intravenous transduction strategy to express GFP in the neurons of *Pfkfb3*^lox/+^ or *CamkIIα-Pfkfb3* mice. HP, hippocampus. Created with https://www.biorender.com (Extended Data Fig. 2f). **d**, Mitochondrial ROS production in neurons isolated from mice transduced with AAV expressing GFP under the neuron-specific hSyn promoter. Data are mean ± s.e.m. *P* values are indicated; *n* = 5 (WT) or *n* = 4 (*CamkIIα-Pfkfb3*, *CamkIIα-mCat*, *CamkIIα-Pfkfb3-mCat*) mice; one-way ANOVA followed by Bonferroni correction (Extended Data Fig. 2f). **e**, Mitochondrial CI activity. Data are mean ± s.e.m. *P* values are indicated; *n* = 6 (WT), *n* = 8 (*Pfkfb3*), *n* = 3 (*mCat*) or *n* = 9 (*Pfkfb3*-*mCat*) biologically independent cell culture preparations; one-way ANOVA followed by Bonferroni correction. **f**, Mitochondrial complex II–III activity. Data are mean ± s.e.m. *n* = 6 (WT, *Pfkfb3*), *n* = 3 (*mCat*) or *n* = 7 (*Pfkfb3*-*mCat*) biologically independent cell culture preparations; one-way ANOVA followed by Bonferroni correction. **g**, In-gel activity of CI (IGA-CI) and BNGE followed by immunoblotting for CI subunits NDUFS1 and NDUFA9, complex (C)III subunit UQCRC2, complex IV subunit MTCO1, complex V subunit ATPβ or complex II subunit SDHA in primary neurons (Extended Data Fig. 2c). **h**, Deactive mitochondrial CI activity. Data are mean ± s.e.m. *P* values are indicated; *n* = 6 (WT, *Pfkfb3*) or *n* = 4 (*mCat*, *Pfkfb3*-*mCat*) biologically independent cell culture preparations; one-way ANOVA followed by Bonferroni correction. **i**, OCR analysis and calculated parameters. Data are mean ± s.e.m. *P* values are indicated; *n* = 4 (WT, *Pfkfb3*) or *n* = 3 (*mCat*, *Pfkfb3*-*mCat*) biologically independent cell culture preparations; one-way ANOVA followed by Bonferroni correction. Ant, antimycin; olig, oligomycin; rot, rotenone. **j**, Mitochondrial membrane potential (∆*ψ*_m_). Data are mean ± s.e.m. *P* values are indicated; *n* = 5 (WT), *n* = 8 (*Pfkfb3*), *n* = 3 (*mCat*) or *n* = 5 (*Pfkfb3*-*mCat*) biologically independent cell culture preparations; one-way ANOVA followed by Bonferroni correction (Extended Data Fig. 2d,f). Source data

**Extended Data Fig. 2:**
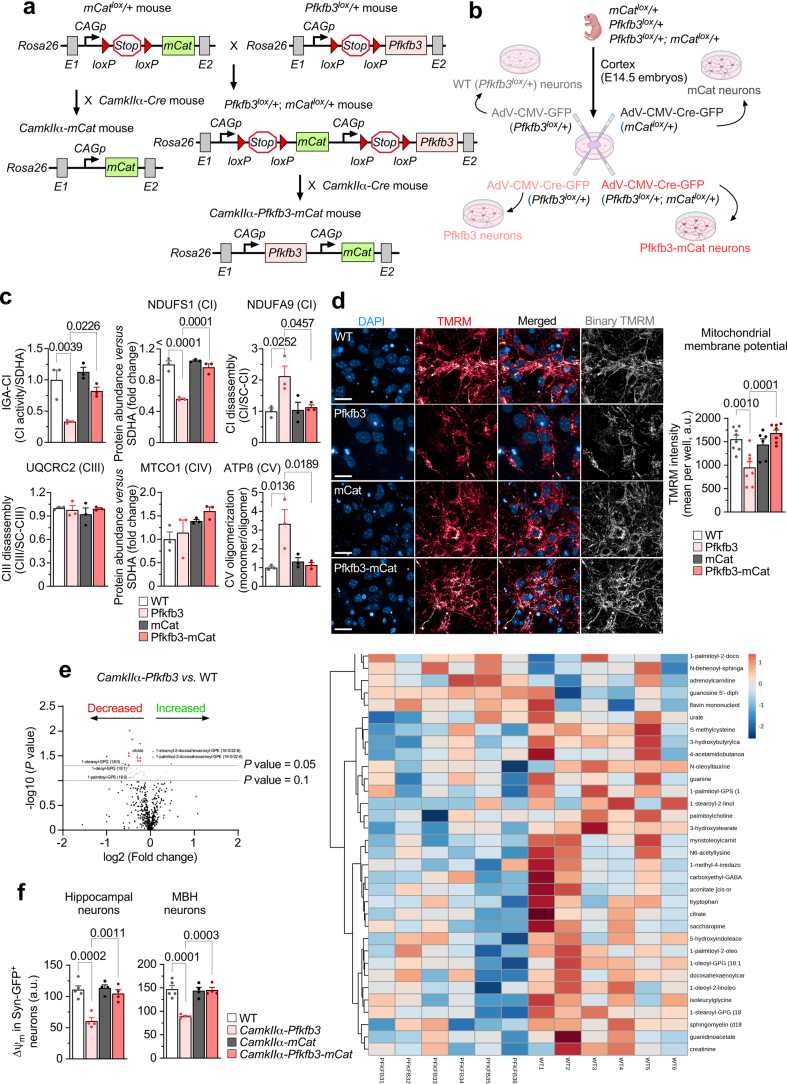
Neuron-specific *Pfkfb3* expression impairs mitochondrial bioenergetics *via* enhanced mitochondrial ROS. **(a)** Strategy used to generate the neuron-specific double transgenic Pfkfb3-mCat mice (*CamkIIα-Pfkfb3-mCat*) and its corresponding control (*CamkIIα-mCat*). **(b)** Adenoviral transduction strategy used to express double transgenic Pfkfb3-mCat in brain cortical neurons in primary culture from *Pfkfb3*^*lox*^*/+; mCat*^*lox*^*/+* mice and its corresponding control (mCat, obtained from *mCat*^*lox*^*/+*). Transduction of *Pfkfb3*^*lox*^*/+* neurons with adenovirus harboring (or not) Cre, generated Pfkfb3 or their corresponding WT (*Pfkfb3*^*lox*^*/+*) neurons. Created with BioRender.com. **(c)** IGA-CI and BNGE quantifications for Fig. 2g. Data are mean ± S.E.M. *P* value is indicated (n = 3 mice per genotype; One-way ANOVA followed by Tukey). (Uncropped western blot replicas are shown in the Source Data file). **(d)** ∆ψ_m_ assessment by confocal analysis in primary neurons. Data are mean ± S.E.M. *P* value is indicated; n = 8 (WT, Pfkfb3-mCat), 7 (Pfkfb3) or 6 (mCat) biologically independent cell culture preparations; one-way ANOVA followed by Bonferroni. Bar=20 µm. **(e)** Volcano plot (left) and heatmap (right) of the metabolomics analysis of the brain of *CamkIIα-Pfkfb3 versus* the corresponding WT mice. Citrate levels (fold-change, mean ± S.E.M.): 0.81 ± 0.0253 (*P* = 0.0345; n = 6 per genotype; Welch’s two-sample *t* test). **(f)** ∆ψ_m_ analysis of hippocampal or MBH neurons freshly isolated from mice of the different genotypes previously transduced with adeno-associated viral particles expressing GFP under the neuron-specific *hSyn* promoter. Data are mean ± S.E.M. *P* value is indicated; n = 5 (WT) or 4 (*CamkIIα-Pfkfb3*, *CamkIIα-mCat, CamkIIα-Pfkfb3-mCat*) mice; one-way ANOVA followed by Bonferroni. Related to Fig. 2d. Source data

Given that GSH is an antioxidant, we next searched for possible redox stress in *Pfkfb3* neurons. Reactive oxygen species (ROS) were thus assessed using two approaches. Using Amplex Red, we observed an increase in fluorescence that was potentiated by rotenone (Fig. 2a), suggesting enhanced ROS by forward electron transfer at mitochondrial complex I (CI). We next used the mitochondrial-specific probe MitoSOX, which revealed increased mitochondrial ROS (Fig. 2b). To further confirm the mitochondrial origin of these ROS, we used a genetic approach to express mitochondrial matrix-tagged catalase (*mCat*^lox/+^)^20^. Mating *Pfkfb3*^lox/+^ mice with *mCat*^lox/+^ mice generated double-transgenic *Pfkfb3*^lox/+^;*mCat*^lox/+^ mice and siblings harbouring either genotype alone, namely, *Pfkfb3*^lox/+^ or *mCat*^lox/+^ (Extended Data Fig. 2a). Littermate embryos were individually genotyped and used to obtain neurons in primary culture (Extended Data Fig. 2b). Transduction of *Pfkfb3*^lox/+^, *mCat*^lox/+^ and *Pfkfb3*^lox/+^;*mCat*^lox/+^ cells with AAV-CMV-Cre-GFP yielded *Pfkfb3*, *mCat* or *Pfkfb3*-*mCat* neurons, respectively (Extended Data Fig. 2b); controls were *Pfkfb3*^lox/+^ cells transduced with AAV-CMV-GFP (Extended Data Fig. 2b). The increases in rotenone-induced Amplex Red and MitoSOX fluorescence signal observed in *Pfkfb3* neurons were abolished in *Pfkfb3-mCat* neurons (Fig. 2a,b), confirming the mitochondrial matrix origin of the ROS signal (mROS). To corroborate these findings in vivo, *CamkIIα-Pfkfb3* mice were intravenously transduced with adenovirus expressing green fluorescent protein (GFP) under the control of the neuron-specific promoter hSyn (AAV-PHP.eb-hSyn-GFP) (Fig. 2c). The hippocampus and the hypothalamus were then dissociated, and GFP^+^ cells were analysed by flow cytometry for MitoSOX fluorescence, the intensity of which was enhanced in both brain areas (Fig. 2d). To abolish mROS in neurons in vivo, double-transgenic *Pfkfb3*^lox^^/+^;*mCat*^lox/+^ mice were mated with *CamkIIα-cre* mice to generate *CamkIIα-Pfkfb3-mCat* mice (Extended Data Fig. 2a). *Pfkfb3*^lox/+^ (wild type) and *CamkIIα-mCat* mice were used as controls for *CamkIIα-Pfkfb3* and *CamkIIα-Pfkfb3-mCat* mice, respectively (Extended Data Fig. 2a). As depicted in Fig. 2d, the increased mROS of neurons from *CamkIIα-Pfkfb3* mice was not observed in *CamkIIα-Pfkfb3-mCat* mice. These data indicate that glycolytically active neurons experience mitochondrial redox stress in vivo.

As excess mROS may cause mitochondrial damage^21^, we next assessed the mitochondrial respiratory chain in *Pfkfb3* neurons. As shown in Fig. 2e, mitochondrial CI activity was impaired in *Pfkfb3* neurons but not in *Pfkfb3*-*mCat* neurons; however, complex II–III was unaffected (Fig. 2f). To further explore this, we determined the mitochondrial respiratory chain (MCR) assembly of mitochondrial proteins by blue native gel electrophoresis (BNGE). *Pfkfb3* neurons showed a selective loss of free CI according to the in-gel activity assay (Fig. 2g and Extended Data Fig. 2c). CI-specific activity analysis in *Pfkfb3* neurons revealed an increase in the deactive^22^ form of the complex, which was not observed in *Pfkfb3*-*mCat* cells (Fig. 2h), indicating that mROS mediates CI deactivation and inhibition. Notably, free CI, that is, the fraction of CI that is not superassembled in supercomplexes, lost its NADH-binding module; however, this module was intact in supercomplex-assembled CI, and the ubiquinone-binding CI module was intact (Fig. 2g and Extended Data Fig. 2c). As these data suggest mitochondrial respiratory chain dysfunction, we next performed bioenergetic profile analysis using Seahorse technology. This revealed an impairment in basal, maximal and ATP-linked oxygen-consumption rates (OCRs) in *Pfkfb3* neurons that was corrected in *Pfkfb3*-*mCat* cells (Fig. 2i). Flow cytometry (Fig. 2j) and confocal microscopy (Extended Data Fig. 2d) analyses showed loss in the inner mitochondrial membrane potential (∆*ψ*_m_) in *Pfkfb3* neurons, an effect that was cancelled by mCat. Metabolomics analysis in the brain of the *CamkIIα-Pfkfb3* mice revealed significant alterations in metabolic pathways, including a reduced concentration of the TCA cycle intermediate citrate (Extended Data Fig. 2e). To confirm bioenergetic failure of neurons in vivo, hippocampal and MBH GFP^+^ cells (Fig. 2c) were analysed by flow cytometry, which showed ∆*ψ*_m_ loss in *CamkIIα-Pfkfb3* mice that was corrected in *CamkIIα-Pfkfb3-mCat* mice (Extended Data Fig. 2f). These data indicate that neurons with active glycolysis undergo mROS-mediated deactivation of mitochondrial CI, causing bioenergetic failure.

**Fig. 3:**
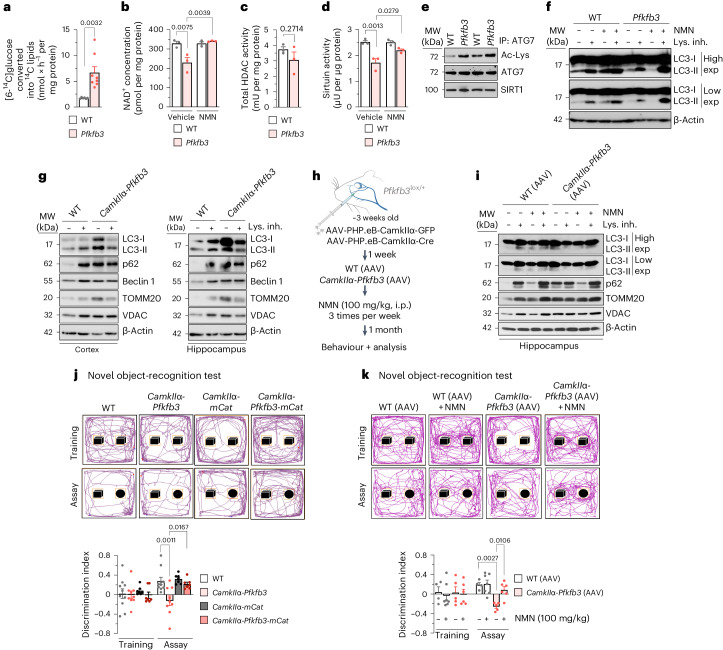
Neuron-specific *Pfkfb3* expression inhibits sirtuin-mediated autophagy, causing motor and cognitive impairment via mitochondrial ROS. **a**–**d**, Lipogenesis rate (**a**), NAD^+^ concentration (**b**), total HDAC activity (**c**) and sirtuin activity (**d**) in primary neurons. Data are mean ± s.e.m. *P* values are indicated; *n* = 6 (**a**, WT), *n* = 8 (**a**, *Pfkfb3*) or *n* = 3 (**b**–**d**) biologically independent cell culture preparations; unpaired Student’s *t*-test, two sided (**a**,**c**); two-way ANOVA followed by Tukey’s test (**b**,**d**). **e**, Immunoprecipitation (IP) of ATG7, followed by western blot for acetyl-lysine (Ac-Lys), ATG7 and sirtuin 1 (SIRT1) in primary neurons; *n* = 3 biologically independent cell culture preparations are shown (Extended Data Fig. 3f). **f**, Autophagic and mitophagic flux analyses by western blot for microtubule-associated proteins 1A/1B light chain 3B (LC3-I and LC3-II) in primary neurons incubated, or not, with NMN or lysosomal inhibitors (lys. inh.). β-Actin, loading control (Extended Data Fig. 3d). Exp, exposure. **g**, Autophagic flux analysis by western blot for LC3-I, LC3-II, p62, beclin 1, mitochondrial import receptor subunit TOM20 homolog (TOMM20) and voltage-dependent anion-selective channel protein (VDAC) in freshly obtained cortical and hippocampal slices from wild-type (WT) or *CamkIIα-Pfkfb3* mice, incubated, or not, with lysosomal inhibitors. β-Actin, loading control (Extended Data Fig. 3e). **h**, In vivo adeno-associated viral intravenous transduction strategy to express GFP or Cre recombinase in neurons of *Pfkfb3*^lox/+^ mice to generate WT (AAV) and *CamkIIα-Pfkfb3* (AAV) mice, respectively. Created with https://www.biorender.com (Extended Data Fig. 3i). i.p., intraperitoneal. **i**, Autophagic and mitophagic flux analyses by western blot for LC3-I, LC3-II, p62, TOMM20 and VDAC in freshly obtained hippocampal slices from WT (AAV) or *CamkIIα-Pfkfb3* (AAV) mice, incubated, or not, with lysosomal inhibitors. β-Actin, loading control (Extended Data Fig. 3k). **j**, Novel object-recognition test in mice of the indicated genotypes. Data are mean ± s.e.m. *P* values are indicated; *n* = 12 (WT), *n* = 9 (*CamkIIα-Pfkfb3*) or *n* = 7 (*CamkIIα-mCat*, *CamkIIα-Pfkfb3-mCat*) mice; one-way ANOVA followed by Bonferroni correction (**h**–**j**) or two-way ANOVA followed by Tukey’s test (**k**). **k**, Novel object-recognition test in mice of the indicated genotypes. Data are mean ± s.e.m. *P* values are indicated; *n* = 4 (WT AAV), *n* = 7 (WT AAV with NMN), *n* = 5 (*CamkIIα-Pfkfb3* AAV) or *n* = 6 (*CamkIIα-Pfkfb3* AAV with NMN) mice; two-way ANOVA followed by Tukey’s test. Source data

**Extended Data Fig. 3:**
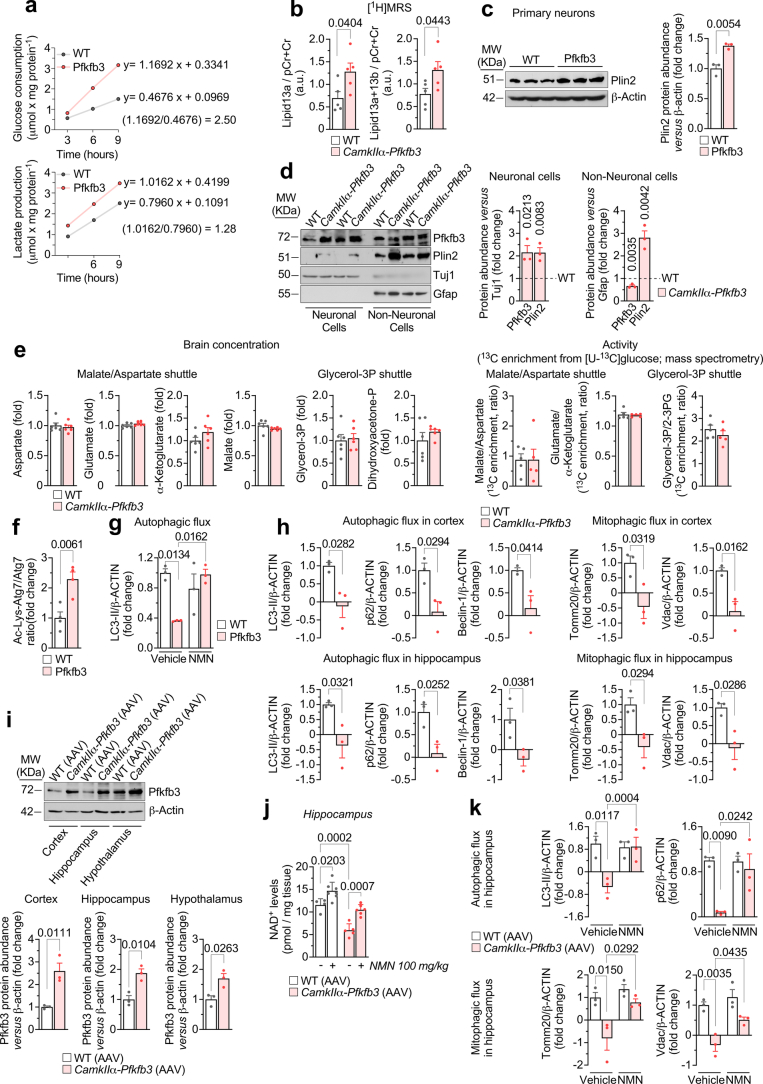
Neuron-specific *Pfkfb3* expression causes increased lipogenesis, lipid droplets accumulation and inhibition of sirtuin-mediated autophagy. **(a)** Rates of glucose consumption and lactate release. Data are averages; 3 replica of a cell culture preparation per condition. **(b)** *In vivo* [^1^H]MRS analysis of lipid13A/(pCr+Cr) and lipid13A + 13B/(pCr+Cr) in mice brain. Data are mean ± S.E.M. *P* value is indicated; n = 5 mice per genotype; Unpaired Student’s *t*-test, two-sided. **(c)** Western blot against perilipin 2 (Plin2). Data are mean ± S.E.M. *P* value is indicated; n = 3 biologically independent cell culture preparations; Unpaired Student’s *t*-test, two-sided. (Uncropped western blot replicas are shown as Source Data file). **(d)** Western blot in brain cells. Gfap and Tuj1, loading controls for astrocytes and neurons, respectively. Data are mean ± S.E.M. *P* value is indicated (n = 3 mice per genotype; Unpaired Student’s *t*-test, two-sided). (Uncropped western blot replicas are shown in the Source Data file). **(e)** Shuttles. 2- + 3-phosphoglycerate (2-3PG) was used as a proxy of dihydroxyacetone-phosphate. Data are mean ± S.E.M. *P* value is indicated (n = 6 mice per genotype; Unpaired Student’s *t*-test, two-sided). (^13^C-enriched metabolites are shown in the Source Data file). **(f-h)** Western blot quantifications for Fig. 3e,f,g. Data are mean ± S.E.M. *P* values are indicated; n = 4 (f), 3 (g), 3 (h) biologically independent cell culture preparations. Unpaired Student’s *t*-test, two-sided (f,h), two-way ANOVA followed by Tukey (g). (Uncropped western blot replicas are shown in the Source Data file). **(i)** Western blot and quantifications. Data are mean ± S.E.M. *P* value is indicated; n = 3 mice per genotype; Unpaired Student’s *t*-test, two-sided. (Uncropped western blot replicas are shown as Source Data file). **(j)** NAD^+^ concentrations after NMN treatment for 1 month. Data are mean ± S.E.M. *P* value is indicated; n = 3 (*WT* AAV), 7 (*WT* AAV + NMN), 5(*CamkIIα-Pfkfb3* AAV) or 6 (*CamkIIα-Pfkfb3* AAV + NMN) mice; two-way ANOVA followed by Bonferroni. **(k)** Western blot quantifications for Fig. 3i. Data are mean ± S.E.M. *P* value is indicated; n = 3 mice per genotype; two-way ANOVA followed by Tukey. (Uncropped western blot replicas are shown as Source Data file). Source data

Cytosolic NAD^+^ regeneration is required for glycolysis^23,24^. However, dysfunctional mitochondria, such as those in *Pfkfb3* neurons, have impaired their ability to regenerate NAD^+^^24,25^. The rates of glucose consumption and lactate production increased by ~2.5-fold and 1.3-fold, respectively (Extended Data Fig. 3a), showing that pyruvate-to-lactate conversion, while discretely increased by *Pfkfb3* expression (Fig. 1d, in vivo), does not wholly account for the high increase in the rate of glucose consumption. This is in good agreement with the occurrence in neurons of lactate dehydrogenase isoform 1 (LDH1)^4,26^, with a low pyruvate-to-lactate conversion rate and, therefore, a weak capacity to regenerate NAD^+^. We then sought to ascertain whether glucose would be transformed into lipids, a process that requires the conversion of pyruvate into citrate, which leaves mitochondria for lipid synthesis^27,28^. [U-^14^C]glucose conversion into [^14^C]lipids in *Pfkfb3* neurons increased (Fig. 3a) at the same rate as glycolysis (Fig. 1h), suggesting that the vast majority of glycolytically consumed glucose is destined for lipids. Interestingly, metabolomics analysis in the brain of these mice revealed data compatible with reduced lipolysis (Extended Data Fig. 2e), and in vivo ^1^H-MRS brain analysis of *CamkIIα-Pfkfb3* mice showed increased lipid 13a and lipid 13b signals (Extended Data Fig. 3b), which denotes enhanced abundance of lipid droplets^29,30^. To confirm this, we assessed by western blotting the levels of perilipin 2 (PLIN2), a lipid droplet-coating protein that limits the access of lipases, favouring the accumulation of lipids^31^. In cultured primary *Pfkfb3* neurons, PLIN2 levels were considerably increased (Extended Data Fig. 3c). Interestingly, in *CamkIIα-Pfkfb3* mice in vivo, PLIN2 only modestly increased in the neuronal fraction (Extended Data Fig. 3d), but it accumulated abundantly in the non-neuronal fraction, which mostly includes glial cells (Extended Data Fig. 3d). These data strongly suggest that *CamkIIα-Pfkfb3* neurons undergo enhanced synthesis of lipids, which may be partially or transiently stored as lipid droplets and destined for reserve or fuel of neighbouring glial cells. Because lipogenesis from glucose is not an NAD^+^-regenerating process, we assessed NAD^+^ levels in neurons. As shown in Fig. 3b, NAD^+^ concentrations were decreased in *Pfkfb3* neurons, suggesting that active glycolysis causes net NAD^+^ consumption. This was confirmed by incubating *Pfkfb3* neurons with the NAD^+^ precursor nicotinamide mononucleotide (NMN), which fully restored NAD^+^ levels (Fig. 3b). This NAD^+^ loss could not be ascribed to intrinsic impairments in the activities of the NAD^+^-regenerating systems, namely the malate–aspartate and glycerol-3-phosphate shuttles, which remained unchanged according to in vivo analyses (Extended Data Fig. 3e). NAD^+^ is an essential substrate for sirtuin deacetylase activity^32^, which therefore should be affected by NAD^+^ loss. Although histone deacetylase (HDAC) activity (reflecting total sirtuin activity) was unaltered (Fig. 3c), sirtuin-specific activity was impaired in *Pfkfb3* neurons, an effect that was rescued by NMN (Fig. 3d). Interestingly, ATG7, a protein that requires sirtuin-mediated deacetylation to promote autophagy^32^, was hyperacetylated (Fig. 3e and Extended Data Fig. 3f), suggesting impaired autophagy in glycolytically active neurons. To test this, we determined the autophagic flux, which was decreased in *Pfkfb3* neurons, an effect that was rescued by NMN (Fig. 3f and Extended Data Fig. 3g). This result was confirmed ex vivo in the cortex and hippocampus freshly dissected from the *CamkIIα-Pfkfb3* brain, where both the rates of autophagy and mitophagy were reduced (Fig. 3g and Extended Data Fig. 3h). To further ascertain whether NAD^+^ restoration corrects these biochemical alterations in vivo, newly weaned *Pfkfb3*^lox/+^ mice were intravenously transduced with adenovirus expressing Cre under the control of the *CamkIIα* promoter (AAV-PHP.eb-CamkIIα-Cre) (Fig. 3h), which efficiently expresses *Pfkfb3* in the cortex, hippocampus and hypothalamus (Extended Data Fig. 3i). One week after, NMN was intraperitoneally administered (100 mg per kg body weight) to these mice at three injections per week for 1 month. As shown in Extended Data Fig. 3j, this in vivo NMN treatment restored the decreased hippocampal NAD^+^ levels observed in mice expressing neuronal *Pfkfb3* and rescued impaired autophagy and mitophagy (Fig. 3i and Extended Data Fig. 3k). These data indicate that glycolytically active neurons show an impaired autophagic flux and that, by modulating the NAD^+^ pool, glycolysis regulates autophagy.

**Extended Data Fig. 4:**
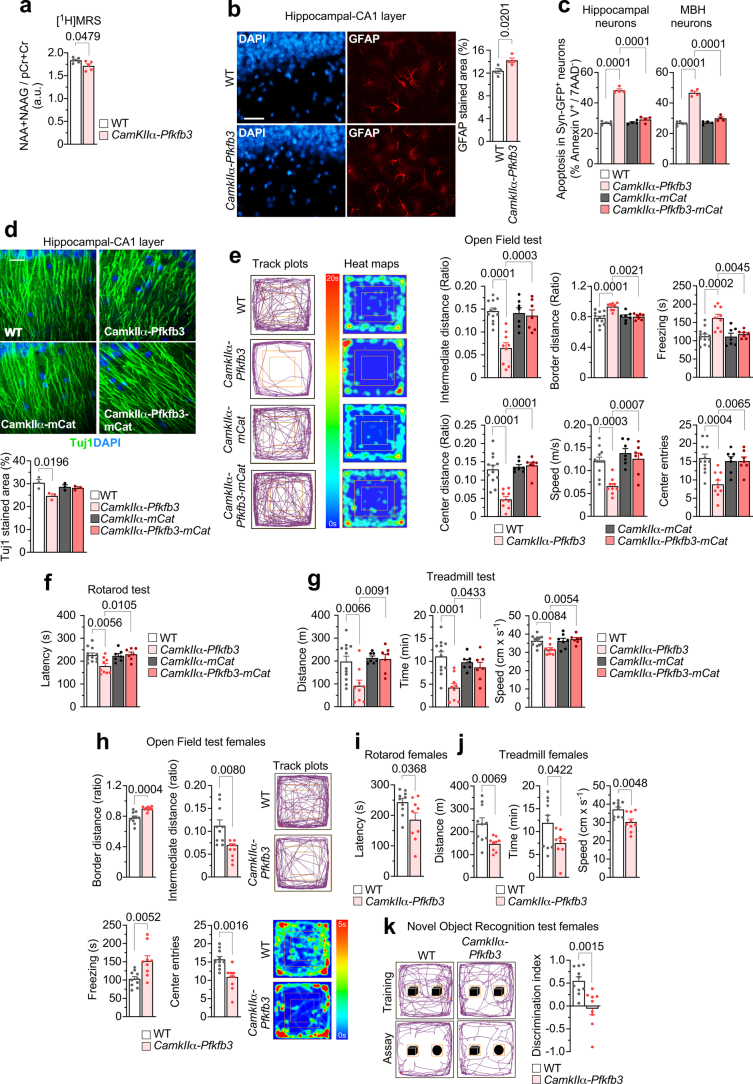
Neuron-specific *Pfkfb3* expression causes motor and cognitive impairments *via* mitochondrial ROS. **(a)** *In vivo* [^1^H]MRS analysis of NAA + NAAG/(pCr+Cr) in the brain of WT and *CamkIIα-Pfkfb3* mice. Data are mean ± S.E.M. *P* value is indicated; n = 5 mice per genotype; Unpaired Student’s *t*-test, two-sided. **(b)** Immunocytochemical analysis of hippocampal slices against GFAP and DAPI from WT and *CamkIIα-Pfkfb3* mice. Data are mean ± S.E.M. *P* value is indicated; n = 4 mice per genotype; Unpaired Student’s *t*-test, two-sided. Bar=20 µm. **(c)** Apoptosis analysis of hippocampal or MBH neurons freshly isolated from mice of the different genotypes previously transduced with adeno-associated viral particles expressing GFP under the neuron-specific *hSyn* promoter. Data are mean ± S.E.M. *P* value is indicated; n = 5 (WT) or 4 (*CamkIIα-Pfkfb3*, *CamkIIα-mCat, CamkIIα-Pfkfb3-mCat*) mice; one-way ANOVA followed by Bonferroni. Related to Fig. 2d. **(d)** Immunocytochemical analysis of hippocampal slices against Tuj1 and DAPI from WT, *CamkIIα-Pfkfb3, CamkIIα-mCat and CamkIIα-Pfkfb3-mCat* mice. Data are mean ± S.E.M. *P* value is indicated; n = 3 mice per genotype; one-way ANOVA followed by Bonferroni. Bar=20 µm. **(e)** Open field test analysis in mice. Data are mean ± S.E.M. *P* value is indicated; n = 12 (WT), 9 (*CamkIIα-Pfkfb3*), 7 (*CamkIIα-mCat, CamkIIα-Pfkfb3-mCat*) mice; one-way ANOVA followed by Bonferroni. Related to Fig. 3. **(f,g)** Rotarod (f) and treadmill (g) tests in mice of the indicated genotypes. Data are mean ± S.E.M. *P* value is indicated; n = 12 (WT), 9 (*CamkIIα-Pfkfb3*) or 7 (*CamkIIα-mCat, CamkIIα-Pfkfb3-mCat*) mice; one-way ANOVA followed by Bonferroni. **(h-k)** Open field (h), rotarod (i), treadmill (j) and novel object recognition (k) tests in female mice of the indicated genotypes. Data are mean ± S.E.M. *P* value is indicated; n = 10 (WT) or 9 (*CamkIIα-Pfkfb3*) mice; Unpaired Student’s *t*-test, two-sided. Source data

**Extended Data Fig. 5:**
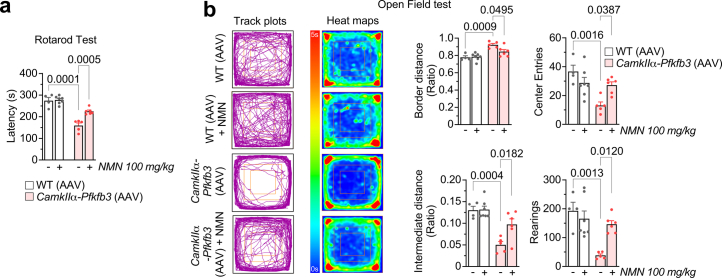
Motor and cognitive impairments caused by neuron-specific *Pfkfb3* expression is rescued by restoring brain NAD^+^ levels. **(a,b)** Rotarod (a) and Open Field (b) tests in mice of the indicated genotypes. Data are mean ± S.E.M. *P* value is indicated; n = 4 (*WT* AAV), 7 (*WT* AAV + NMN), 5 (*CamkIIα-Pfkfb3* AAV) or 6 (*CamkIIα-Pfkfb3* AAV + NMN) mice; two-way ANOVA followed by Tukey. Source data

Next, we aimed to investigate whether the metabolic alterations occurring in glycolytically active neurons impacted their function and viability. According to the brain [^1^H]MRS analysis (Extended Data Fig. 4a), the neuronal functional markers *N*-acetylaspartate and its glutamyl derivative *N*-acetylaspartylglutamate were decreased in *CamkIIα-Pfkfb3* mice, which also showed astrogliosis as determined by glial fibrillary acidic protein (GFAP) staining (Extended Data Fig. 4b). Moreover, flow cytometry analysis of freshly isolated neurons from the hippocampus and hypothalamus of *CamkIIα-Pfkfb3* mice previously transduced with AAV-PHP.eb-hSyn-GFP revealed increased apoptotic neurons (Extended Data Fig. 4c). Moreover, immunocytochemical analysis of the *CamkIIα-Pfkfb3* hippocampus showed disruption of neuronal integrity, as demonstrated by βIII-tubulin (TUJ1) staining (Extended Data Fig. 4d). Notably, these alterations were corrected in *CamkIIα-Pfkfb3-mCat* mice (Extended Data Fig. 4c,d). These set of in vivo data confirm that glycolytic neurons undergo mitochondrial redox stress that contributes to neuronal dysfunction and damage. To address whether the metabolic and functional alterations that take place in glycolytically active neurons impact on the organismal level, we performed a range of behavioural tests. The open-field test (Extended Data Fig. 4e) revealed fear and/or anxiety in *CamkIIα-Pfkfb3* mice. These mice performed worse at the rotarod (Extended Data Fig. 4f) and treadmill (Extended Data Fig. 4g), indicating loss of motor activity, poor endurance and slowness. Furthermore, the novel object-recognition test showed short-term cognitive impairment (Fig. 3j). Female mice showed an identical phenotype (Extended Data Fig. 4h–k). Notably, these alterations were abolished in *CamkIIα-Pfkfb3-mCat* male mice (Fig. 3j and Extended Data Fig. 4e–g). Interestingly, behavioural alterations including motor discoordination, fear and/or anxiety and cognitive impairment were corrected by restoring brain NAD^+^ levels in NMN-treated *Pfkfb3*-expressing mice (Fig. 3k and Extended Data Fig. 5a,b). Thus, the transformation of neurons into glycolytically active cells develops functional alterations in neurons and loss of cognitive performance.

**Fig. 4:**
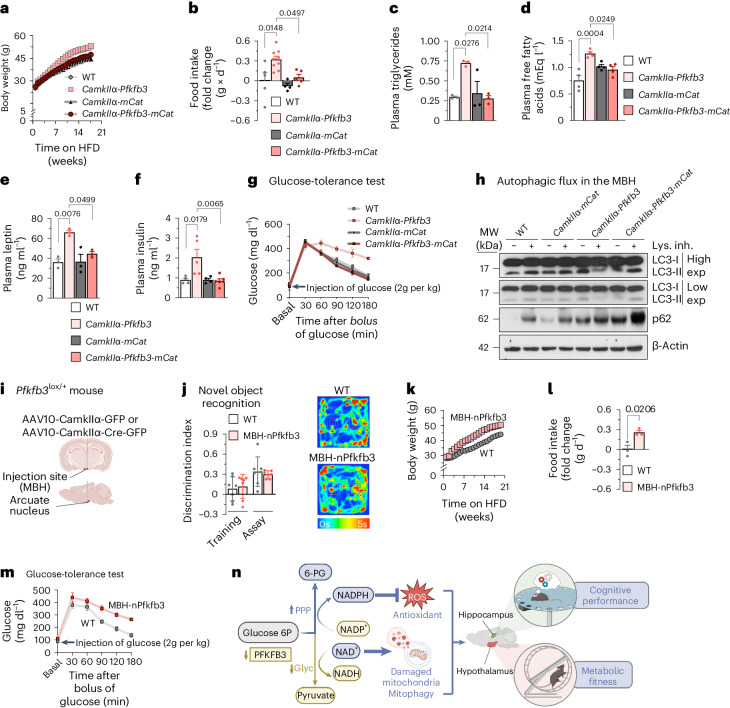
Neuronal *Pfkfb3* induces an mROS-mediated metabolic-like syndrome that is mimicked by confining *Pfkfb3* expression to MBH neurons. **a**, Body weight progression in mice of the indicated genotypes fed the HFD (Extended Data Fig. 6b). **b**–**f**, Food intake (**b**), plasma triglycerides (**c**), free fatty acids (mEq, milliequivalents) (**d**), leptin (**e**) and insulin (**f**) in HFD-fed mice. Data are mean ± s.e.m. *P* values are indicated; *n* = 5 (**b**), *n* = 3 (**c**,**e**) or *n* = 4 (**d**,**f**) for WT mice; *n* = 9 (**b**), *n* = 3 (**c**,**e**) or *n* = 4 (**d**,**f**) for *CamkIIα-Pfkfb3* mice; *n* = 4 (**b**), *n* = 3 (**c**,**e**) or *n* = 4 (**d**,**f**) for *CamkIIα-mCat* mice*;* *n* = 5 (**b**), *n* = 3 (**c**,**e**), *n* = 4 (**d**) or *n* = 6 (**f**) for *CamkIIα-Pfkfb3-mCat* mice; one-way ANOVA followed by Bonferroni correction. **g**, Glucose-tolerance test in HFD-fed mice (Extended Data Fig. 6f). **h**, Autophagic flux analysis by western blot for LC3-I, LC3-II and p62 in freshly obtained MBH slices from mice of the indicated genotypes, incubated, or not, with lysosomal inhibitors. β-Actin was used as the loading control (Extended Data Fig. 6g). **i**, Strategy used to express *Pfkfb3* in MBH neurons by stereotaxic injections of AAV expressing, or not, Cre recombinase. Created with https://www.biorender.com. **j**, Novel object-recognition test analysis in WT and MBH-nPfkfb3 mice. Data are mean ± s.e.m., *n* = 7; unpaired Student’s *t*-test, two sided. **k**, Body weight progression in WT or MBH neurons in *Pfkfb3*-expressing (MBH-nPfkfb3) mice fed the HFD (Extended Data Fig. 6i). **l**, Food intake in WT or MBH-nPfkfb3 mice fed the HFD. Data are mean ± s.e.m. *P* value is indicated (*n* = 3 mice per condition; unpaired Student’s *t*-test, two sided). **m**, Glucose-tolerance test in WT or MBH-nPfkfb3 mice fed the HFD (Extended Data Fig. 6l). **n**, Graphical summary describing the main message of this work. In neurons, low PFKFB3 abundance keeps glycolysis (Glyc) attenuated (which preserves NAD^+^-dependent mitophagy) and boosts the PPP (which regenerates NADPH(H^+^) to prevent redox stress). These mechanisms acting on the hippocampus and on the MBH are required to promote cognition and organismal fitness, respectively. Glucose 6P, glucose 6-phosphate; 6-PG, 6-phosphogluconate. Created with https://www.biorender.com. Source data

**Extended Data Fig. 6:**
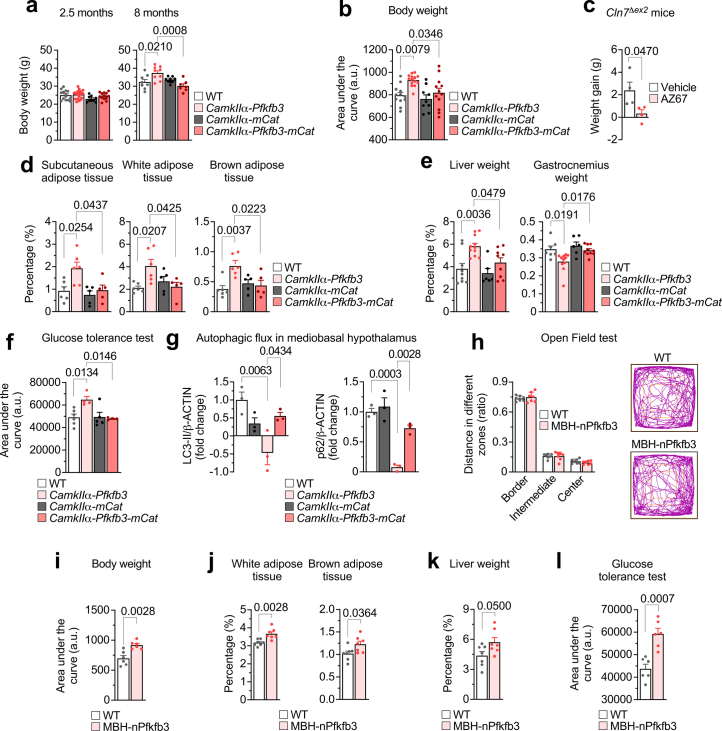
Neuronal *Pfkfb3* induces a mROS-mediated metabolic-like syndrome that is mimicked by confining Pfkfb3 expression in mediobasal hypothalamic neurons. **(a)** Body weight analysis in mice. Data are mean ± S.E.M. *P* value is indicated; for 2.5 months, n = 14 (WT), 20 (*CamkIIα-Pfkfb3*), 9 (*CamkIIα-mCat*) or 13 *(CamkIIα-Pfkfb3-mCat*) mice; for 8 months, n = 8 (WT), 9 (*CamkIIα-Pfkfb3*, *CamkIIα-mCat*) or 7 *(CamkIIα-Pfkfb3-mCat*) mice; one-way ANOVA followed by Bonferroni. **(b)** Analysis of the area under the curve for Fig. 4a. Data are mean ± S.E.M. *P* value is indicated; n = 12 (WT), 14 (*CamkIIα-Pfkfb3*), 9 (*CamkIIα-mCat*) or 12 *(CamkIIα-Pfkfb3-mCat*) mice; one-way ANOVA followed by Tukey. **(c)** Weight gain in *Cln7*^*∆ex2*^ and WT mice that daily received the Pfkfb3 inhibitor AZ67 (1 nmol/day) (or DMSO) intracerebroventricularly for 2.5 months. Data are mean ± S.E.M. *P* value is indicated; n = 4 mice per genotype; Unpaired Student’s *t*-test, two-sided. **(d)** Weight of the indicated tissues in mice. Data are mean ± S.E.M. *P* value is indicated; n = 6 (WT, *CamkIIα-Pfkfb3*) or 5 (*CamkIIα-mCat*, *CamkIIα-Pfkfb3-mCat*) mice; one-way ANOVA followed by Bonferroni. **(e)** Weight of the indicated tissues in mice. Data are mean ± S.E.M. *P* value is indicated; liver, n = 9 (WT), 11 (*CamkIIα-Pfkfb3*), 6 (*CamkIIα-mCat*) or 9 *(CamkIIα-Pfkfb3-mCat*) mice; gastrocnemius, n = 7 (WT), 11 (*CamkIIα-Pfkfb3*), 6 (*CamkIIα-mCat*) or 10 *(CamkIIα-Pfkfb3-mCat*) mice; one-way ANOVA followed by Tukey. **(f)** Analysis of the area under the curve of glucose tolerance test of Fig. 4g. Data are mean ± S.E.M. *P* value is indicated; n = 6 (WT), 4 (*CamkIIα-Pfkfb3*), 5 (*CamkIIα-mCat*) or 4 (*CamkIIα-Pfkfb3-mCat*) mice; one-way ANOVA followed by Tukey. **(g)** Western blot quantifications for Fig. 4 h. Data are mean ± S.E.M. *P* value is indicated; n = 3 mice per genotype; one-way ANOVA followed by Tukey. (Uncropped western blot replicas are shown in the Source Data file). **(h)** Open field test analysis in WT and MBH-nPfkfb3 mice. Data are mean ± S.E.M. n = 7. Unpaired Student’s *t*-test, two-sided. **(i-l)** Data are mean ± S.E.M. *P* value is indicated; n = 6 (j, m) or 7 (k, l) mice per genotype; Unpaired Student’s *t*-test, two-sided. (l, related to Fig. 4m). Source data

To further characterize mice with glycolytically active neurons, we monitored weight. *CamkIIα-Pfkfb3* mice showed no weight change at 2.5 months, but body weight increased at the age of 8 months (Extended Data Fig. 6a). To boost this phenotype, mice were fed a high-fat diet (HFD) from weaning for 21 weeks. As shown in Fig. 4a and Extended Data Fig. 6b, the weight of *CamkIIα-Pfkfb3* mice progressively increased, along with food intake (Fig. 4b), effects that were abolished in *CamkIIα-Pfkfb3-mCat* mice. In the neuronal ceroid lipofuscinosis *Cln7*^∆ex2^ mouse model, we previously observed neuronal PFKFB3 protein stabilization^10^. Here, we show that chronic (2.5 months, daily) intracerebroventricular administration of the PFKFB3-specific inhibitor AZ67 (ref. ^33^) in *Cln7*^∆ex2^ mice prevented the increase in weight gain observed in vehicle-treated *Cln7*^∆ex2^ mice (Extended Data Fig. 6c). Subcutaneous and abdominal white fat and brown fat (Extended Data Fig. 6d) were increased in *CamkIIα-Pfkfb3* mice but not in *CamkIIα-Pfkfb3-mCat* animals. Likewise, these mice revealed hepatomegaly and sarcopenia (Extended Data Fig. 6e). Levels of plasma triglycerides (Fig. 4c), free fatty acids (Fig. 4d), leptin (Fig. 4e) and insulin (Fig. 4f) were increased in *CamkIIα-Pfkfb3* mice and abolished in *CamkIIα-Pfkfb3-mCat* mice. Finally, *CamkIIα-Pfkfb3* mice were unable to reduce plasma glucose levels in the glucose overload-tolerance test (Fig. 4g and Extended Data Fig. 6f), whereas *CamkIIα-Pfkfb3-mCat* mice performed correctly in this test. Given that these observations suggest metabolic-like syndrome, we assessed the autophagy capacity of the mediobasal hypothalamus (MBH), as this process has been previously reported to be specifically impaired in the MBH^34^. Analysis of the MBH freshly dissected from *CamkIIα-Pfkfb3* mice revealed impaired autophagic flux (Fig. 4h and Extended Data Fig. 6g), an effect that was ablated in *CamkIIα-Pfkfb3-mCat* mice. To assess whether the anatomical origin of this phenotype is the MBH or whether it is the indirect consequence of impaired neuronal circuitry from other brain areas, we confined *Pfkfb3* expression to MBH neurons. Thus, *Pfkfb3*^lox/+^ mice were stereotaxically injected with adenovirus harbouring Cre recombinase governed by the *CamkIIα* promoter (AAV-CamkIIα-Cre-GFP) in the arcuate nucleus of the MBH to induce *Pfkfb3* expression in the neurons of this brain area (MBH-nPfkfb3) (Fig. 4i). At the age of 21 weeks, no alterations in behavioural tests such as the open-field test (Extended Data Fig. 6h) and the novel object-recognition test (Fig. 4j) were observed, indicating lack of fear and anxiety, motor discoordination or cognitive disturbances. However, weight (Fig. 4k and Extended Data Fig. 6i) and food intake (Fig. 4l) increased in MBH-nPfkfb3 mice when compared with mice injected with AAV lacking Cre recombinase (AAV-CamkIIα-GFP). Moreover, these effects were accompanied by increased weight in white and brown (Extended Data Fig. 6j) adipose tissue, hepatomegaly (Extended Data Fig. 6k) and glucose intolerance (Fig. 4m and Extended Data Fig. 6l). Altogether, these results suggest that glycolytically active neurons of the arcuate nucleus impair hypothalamic control of food intake and organismal metabolism. The connection between metabolic-like syndrome and cognitive impairment upon aberrant neuronal glycolysis described here may open new therapeutic perspectives (Fig. 4n).

In conclusion, here we show that neurons actively preserve a persistent hypoglycolytic metabolism to boost the PPP, therefore avoiding redox stress-mediated mitochondrial impairment. Given the inefficiency of LDH1 (refs. ^4,26^), functional mitochondria are essential to regenerate cytosolic NAD^+^ (ref. ^35^) via the malate and glycerol phosphate shuttles^36^, the activities of which remain intrinsically unaltered when transformed in glycolytically active cells. However, in these neurons, mitochondrial function is impaired, thus hindering their ability to regenerate NAD^+^ regardless of shuttle activities, leading to autophagy impairment and cognitive deficiency (Fig. 4n). This mechanism could explain the pathogenic consequences of neuronal PFKFB3 stabilization^9,10^ and the aberrant hyperglycolysis^11^ taking place in Alzheimer’s disease and other disease conditions. Moreover, we show that a similar pattern of events operates in *Pfkfb3*-expressing neurons of the MBH and triggers metabolic-like syndrome (Fig. 4n). Naturally, neuronal glycolysis is amenable to transient activation during glutamatergic neurotransmission^25,37,38^ or hypoxia^39^, where this pathway has energetic functions in specific subcellular compartments^40–43^. However, when compared with neurons, adjacent astrocytes robustly consume glucose via glycolysis^44^ and fatty acids via β-oxidation^45^, generating lactate and ketone bodies, respectively, which can contribute to satisfying neuronal energy needs^2,46^. It is therefore tempting to speculate that the neurological benefits of the ketogenic diet^47^ rely on its ability to sustain the energy demands of weak glycolytic neurons. Thus, neurons are metabolically inflexible cells unable to adapt to the sustained active glycolysis that is linked with neurological and metabolic syndromes, in which neuron-specific inhibition of PFKFB3 may be considered as a therapeutic opportunity.

## Methods

### Mice

All protocols were performed according to the European Union Directive 86/609/EEC and Recommendation 2007/526/EC, regarding the protection of animals used for experimental and other scientific purposes, enforced in Spanish legislation under the law 6/2013. Protocols were approved by the Bioethics Committee of the University of Salamanca. All mice used in this study were of the C57BL/6J background. Both male and female mice were used, although most data shown correspond to male mice unless otherwise stated. Mice were bred at the Animal Experimentation Facility of the University of Salamanca in cages (maximum of five animals per cage), and a light–dark cycle was maintained for 12 h. Humidity was 45–65%, and temperature was 20–25 °C. Animals were fed ad libitum with a standard solid diet (Envigo-Harlan Teklad Global 18% Protein Rodent Diet; 18% protein, 3% lipids, 58.7% carbohydrate component, 4.3% cellulose, 4% minerals and 12% humidity) and had free access to water. When indicated, animals were switched to an HFD (D12451, Research Diets; 20% protein, 45% lipids and 35% carbohydrates, plus minerals and vitamins).

### In vivo generation of neuron-specific *Cdh1*^−/−^ mice

To inactivate the *Cdh1* gene in neurons of the adult brain, *Cdh1*^lox/lox^ mice^12^ were mated with mice carrying the gene encoding Cre recombinase under the control of the *CamkIIα* promoter (Jackson Laboratory)^13^, generating *CamkIIα-Cdh1*^−/−^ mice.

### In vivo generation of neuron-specific *Pfkfb3*-expressing mice

*Pfkfb3*^lox/+^ mice were generated by homologous recombination in the *Rosa26* locus of embryonic stem cells of the C57BL/6J background, where we introduced the full-length cDNA for mouse *Pfkfb3* preceded by a transcriptional stop cassette flanked by two *loxP* sites. This *loxP*-flanked stop signal was incorporated between the CAG promoter and the mouse *Pfkfb3* cDNA (Fig. 1a). To express *Pfkfb3* in neurons in vivo, *Pfkfb3*^lox^^/+^ mice were mated with *CamkIIα-cre* mice to generate *CamkIIα-Pfkfb3* mice. Where indicated, neuron-specific *Pfkfb3*-expressing mice were generated by intravenously transduced adenovirus expressing Cre under control of the *CamkIIα* promoter (AAV-PHP.eB-CamkIIα-Cre) in weaned (~3-week-old) *Pfkfb3*^lox/+^ mice; controls were *Pfkfb3*^lox/+^ mice transduced with a virus expressing GFP instead of Cre (AAV-PHP.eB-CamkIIα-GFP).

### In vivo generation of neuron-specific *mCat-Pfkfb3*-expressing mice

We first mated *mCat*^lox/+^ mice^20^ with *Pfkfb3*^lox/+^ mice, which generated double-transgenic *Pfkfb3*^lox/+^;*mCat*^lox/+^ mice. Next, *Pfkfb3*^lox^^/+^;*mCat*^lox/+^ mice were mated with *CamkIIα-cre* mice to generate *CamkIIα-Pfkfb3-mCat* mice.

### Treatment of mice with nicotinamide mononucleotide

NMN was intraperitoneally administered (100 mg per kg body weight; 200 µl per animal dissolved in phosphate-buffered saline (PBS) as the vehicle) to ~4-week-old mice at three injections per week for 1 month. Control mice received an identical volume of the vehicle.

### Neurons in primary culture

Neurons in primary culture were prepared from the cortex of E14.5 embryos^20^ of the genotypes *Pfkfb3*^lox/+^, *mCat*^lox/+^ and *Pfkfb3*^lox/+^;*mCat*^lox/+^. Littermates were genotyped and used individually. Cell suspensions were seeded at 2.0 × 10^5^ cells per cm^2^ in poly-d-lysine (10 μg ml^−1^)-coated plastic plates in Neurobasal-A medium supplemented with 2 mM glutamine, 5.5 mM glucose, 0.22 mM pyruvate and 2% antioxidant B27 supplement. Cells were incubated at 37 °C in a humidified 5% CO_2_-containing atmosphere. Seventy-two hours after plating, the medium was replaced with medium containing 2% of the minus-antioxidant (that is, lacking vitamin E, vitamin E acetate, superoxide dismutase, catalase and glutathione) B27 supplement, and cells were transduced with AAV-CMV-Cre-GFP or AAV-CMV-GFP to obtain transgene expression or controls, as indicated, and further incubated with fresh medium until day 7. Immunocytochemical analysis revealed that ~99.5% of cells were neurons^48^.

### Genotyping by polymerase chain reaction

For *Pfkfb3*^lox/+^ genotyping, a PCR with the following primers was performed: 5′-CGTGATCTGCAACTCCAGTCTTTC-3′, 5′- CCCAGATGACTACTTATCCTCCCA-3′ and 5′-TCCCAGTCATAGCTGTCCCTCTTC-3′. PCR conditions were 5 min at 98 °C, 25 cycles of 5 s at 98 °C, 5 s at 60 °C, 20 s at 72 °C and a final extension of 2 min at 72 °C, resulting in an 82-bp band for *Pfkfb3*^lox/lox^ mice and a 217-bp band for wild-type mice. Primers for genotyping the *mCat* allele were 5′-CTCCCAAAGTCGCTCTGAGTTGTTATCA-3′, 5′-CGATTTGTGGTGTATGTAACTAATCTGTCTGG-3′ and 5′-GCAGTGAGAAGAGTACCACCATGAGTCC-3′, which yielded a 778-bp band for the wild-type allele and a 245-bp band for the *mCat* allele. PCR conditions were 5 min at 94 °C, 35 cycles of 30 s at 94 °C, 30 s at 65 °C, 3 min at 68 °C and 8 min at 68 °C. PCR products were resolved on a 3% agarose gel using the 1 Kb Plus DNA ladder (Thermo Fisher Scientific).

### Immunomagnetic purification of neurons and astrocytes from the adult brain

The brain (without the cerebellum and the olfactory bulb) was dissociated using the adult mouse brain dissociation kit (Miltenyi Biotec). The tissue, once clean, was fragmented with a sterile scalpel in 2 ml per hemisphere of a disintegration solution (Earle’s Balanced Salt Solution, 116 mM NaCl, 5.4 mM KCl, 1.5 mM MgSO_4_, 26 mM NaHCO_3_, 1.01 mM NaH_2_PO_4_·2H_2_O, 4 mM glucose, 10 mg l^−1^ phenol red, supplemented with 14.4 μl ml^−1^ albumin and 26 μl ml^−1^ DNase type I, pH 7.2), and it was trypsinized (10.8 μl ml^−1^ trypsin) at 37 °C in a thermostated bath for 5 min, shaking frequently to avoid decantation of the tissue. Next, the suspension was triturated using a 5-ml serological pipette (five times) and further incubated for 10 min with frequent shaking. Trypsin activity was stopped by adding 10% foetal calf serum, before centrifuging the tissue at 700*g* for 5 min in a microcentrifuge at 4 °C. Once the enzymatically disintegrated tissue was decanted, the pellet was resuspended in a trypsin-free disintegration solution (Earle’s Balanced Salt Solution with 13 μl ml^−1^ DNase and 20 μl ml^−1^ albumin) for trituration using a fire-polished Pasteur pipette. Approximately five passages were performed for a volume of 4 ml per hemisphere. The supernatant was centrifuged for 3 min at 700*g*, and cells in the pellet were counted. Once a homogeneous suspension of individualized adult neural cells was achieved, cell population separation was performed using MACS Technology with either the astrocyte-specific Anti-ACSA-2 MicroBead Kit or the neuron-specific Neuron Isolation Kit, according to the manufacturer’s protocol (MACS Technology). We confirmed the identity of the isolated fractions by western blotting for neuronal (TUJ1)- or astrocytic (GFAP)-specific markers^48^.

### In vivo viral transduction

This was carried out using a validated AAV strategy^49^. Essentially, AAV particles of the PHP.eB capsid (serotype), known to efficiently transduce the central nervous system via intravenous injection^50^ and expressing Cre recombinase driven by the neuron-specific hSyn promoter (AAV-PHP.eB-hSyn-Cre-GFP), were administered intravenously (50-µl aliquots of a PBS solution containing 0.001% Pluronic F-68, Sigma-Aldrich and 5 × 10^10^ viral genomes) through the retro-orbital sinus to 2-month-old *Pfkfb3*^lox/+^ mice under brief sevoflurane anaesthesia (Sevorane, Abbot, at 6% for initiation followed by ~3% for maintenance in air with supplementary O_2_ and NO_2_ at 0.4 and 0.8 l min^−1^, respectively, using a gas distribution column (Hersill H-3) and a vaporiser (InterMed Penlon Sigma Delta). We used the retro-orbital sinus intravenous route because of the higher success rate observed when compared with the tail or temporal ones^51^. To obtain controls, *Pfkfb3*^lox/+^ siblings received equivalent amounts of the same AAV particles that did not harbour Cre recombinase. Mice were used from 4 weeks after AAV injections.

### Stereotaxic injections

Nine-week-old *Pfkfb3*^lox/+^ mice were anaesthetized by inhalatory induction (4%) and maintained (2.5%) with sevoflurane (Sevorane, Abbot) in a gas mixture of 70% N_2_O and 30% O_2_, using a gas distribution column (Hersill H-3) and a vaporiser (InterMed Penlon Sigma Delta). Mice were placed in a stereotaxic alignment system (model 1900, David Kopf Instruments) complemented with a stereomicroscope (Nikon SMZ645) and a fibre optic cold light source (Schott KL1500 compact). Injection was performed into the arcuate nucleus at coordinates AP = −2.00 mm, ML = +0.25 mm and DV = −5.5 mm from the bregma using a 5-μl Hamilton syringe with a (Microliter 65RN) 26S needle (type 2 tip). Either 0.5 μl of AAV10-CamkIIα-Cre-GFP or AAV10-CamkIIα-GFP (2.75 × 10^12^ viral genomes) was injected using a 5-µl Hamilton syringe at a rate of 0.25 µl min^−1^ during 2 min with a mini-pump (UltraMicroPump III, World Precision Instruments). The same volume of the dye Evans blue (Sigma) was injected in the same manner to confirm the injection site. At the end of the injection, the syringe was left in place for 5 min before being slowly removed to prevent reflux. The skin was sutured, and mice were allowed to recover in a warming cabinet (Plactronic Digital, 25 × 60, JP Selecta).

### Mouse perfusion, immunohistochemistry and image analysis

Animals were deeply anaesthetized by intraperitoneal injection of a mixture (1:4) of xylazine hydrochloride (Rompun, Bayer) and ketamine hydrochloride–chlorbutol (Imalgene, Merial) using 1 ml of the mixture per kg body weight and then perfused intra-aortically with 0.9% NaCl followed by 5 ml per g body weight of Somogy’s fixative (4% (wt/vol) paraformaldehyde, 0.2% (wt/vol) picric acid in 0.1 M phosphate buffer, pH 7.4). After perfusion, brains were dissected out sagittally in two parts and postfixed, using Somogy’s fixative, for 2 h at room temperature. Brain blocks were then rinsed successively for 10 min, 30 min and 2 h with 0.1 M PBS (pH 7.4) and sequentially immersed in 10%, 20% and 30% (wt/vol) sucrose in PBS until they sank. After cryoprotection, 10-, 20- and 40-mm-thick sagittal sections were obtained with a freezing–sliding cryostat (Leica, CM1950 AgProtect).

Sections were rinsed with 0.1 M PBS three times each for 10 min and then incubated with (1) 1:1,000 anti-NeuN (A-60, Merck Millipore), 1:300 anti-TUJ1 (T2200, Sigma), 1/500 anti-GFAP (G6171, Sigma), 1/100 anti-PFKFB3 (H00005209-M08, Novus Biologicals), 1/500 anti-cyclin B1 (sc-7393, Santa Cruz Biotechnology), 1/500 anti-ROCK2 (sc-398519, Santa Cruz Biotechnology) antibodies in 0.2% Triton X-100 (Sigma-Aldrich) and 5% goat serum (Jackson ImmunoResearch) in 0.1 M PBS for 72 h at 4 °C and (2) the fluorophore-conjugated secondary antibodies goat anti-rabbit-Cy2 (111-225-144, Jackson ImmunoResearch, 1/500) or goat anti-mouse-Cy5 (115-175-003, Jackson ImmunoResearch, 1/500) in 0.05% Triton X-100 and 2% goat serum in 0.1 M PBS for 2 h at room temperature^14^. After rinsing with PBS, sections were mounted with Fluoromount (Sigma) aqueous mounting medium. Confocal images were taken with a scanning laser confocal microscope (‘Spinning Disk’ Roper Scientific Olympus IX81) with three lasers (405, 491 and 561 nm) and equipped with a ×63 PL Apo oil-immersion objective for high-resolution imaging and a device digital camera (Evolve, Photometrics).

Dendrite integrity in the hippocampus was assayed by analysing the density of TUJ1-positive dendrites in three sections per animal. Fluorescence eight-bit images were acquired as *z* stacks and were exported into ImageJ in TIFF format for processing. Images were converted to greyscale eight-bit images, and brightness and contrast were adjusted using the ImageJ ‘auto’ function. All TUJ1-positive dendrites and GFAP-positive cells were automatically delineated using the ‘auto setting threshold’ (default method) and ‘dark background’ functions of ImageJ. Thresholded images were subsequently quantified as percent area (area fraction) using the ‘analyze-measure’ function, which represents the percentage of pixels in the image that have been highlighted (percentage area)^14^. Values are mean ± s.e.m. from 15 measurements.

### Terminal deoxynucleotidyl transferase dUTP nick end-labelling assay

The terminal deoxynucleotidyl transferase dUTP nick end-labelling (TUNEL) assay was performed on 20-μm brain sections, following the manufacturer’s protocol (Roche Diagnostics). Brain sections, fixed as above, were pre-incubated in TUNEL buffer containing 1 mM CoCl_2_, 140 mM sodium cacodylate and 0.3% Triton X-100 in 30 mM Tris buffer, pH 7.2, for 30 min. After incubation at 37 °C with the TUNEL reaction mixture containing terminal deoxynucleotidyl transferase (800 U ml^−1^) and a nucleotide mixture (1 μM) for 90 min, sections were rinsed with PBS and counterstained with Cy3–streptavidin (Jackson ImmunoResearch Laboratories)^52^.

### Plasma determination of lipids and hormones

For serum triglyceride (T2449, Sigma-Aldrich) and fatty acid (NEFA-HR, Wako Pure Chemical) analyses, we used 10 μl of undiluted serum; for insulin (A05105, SPI Bio) and leptin (A05176, SPI Bio) levels, the serum was diluted four times and we used 50 μl and 100 μl, respectively. We used the protocols according to the manufacturer’s instructions.

### Glucose-tolerance test

This analysis was performed by intraperitoneally injecting d-glucose at 2 g per kg body weight (Sigma) into mice that had been previously fasted for 16 h. Blood was collected from tail bleeds every 30-min to 3-h period, and the amount of plasma glucose was determined using a glucometer (FreeStyle Optium Neo, Abbot). Area-under-the-curve values were determined using GraphPad Prism 8 software.

### Mitochondrial reactive oxygen species

Mitochondrial ROS levels were determined with the fluorescent probe MitoSOX (Life Technologies). Cultured cells or adult brain cell suspensions were incubated with 2 μM MitoSOX for 30 min at 37 °C in a 5% CO_2_ atmosphere in HBSS buffer (134.2 mM NaCl, 5.26 mM KCl, 0.43 mM KH_2_PO_4_, 4.09 mM NaHCO_3_, 0.33 mM Na_2_HPO_4_·2H_2_O, 5.44 mM glucose, 20 mM HEPES and 20 mM CaCl_2_·2H_2_O, pH 7.4). The cells were then washed with PBS (136 mM NaCl, 2.7 mM KCl, 7.8 mM Na_2_HPO_4_·2H_2_O, 1.7 mM KH_2_PO_4_, pH 7.4) and collected by trypsinization. MitoSOX fluorescence intensity was assessed by flow cytometry (FACSCalibur flow cytometer, BD Biosciences) and expressed in AU.

### Hydrogen peroxide determination

For H_2_O_2_ assessments, Amplex Red (Life Technologies) was used. Cells were trypsinized and incubated in KRPG buffer (145 mM NaCl, 5.7 mM Na_2_HPO_4_, 4.86 mM KCl, 0.54 mM CaCl_2_, 1.22 mM MgSO_4_, 5.5 mM glucose, pH 7.35) in the presence of 9.45 μM Amplex Red containing 0.1 U ml^−1^ horseradish peroxidase. Luminescence was recorded for 2 h at 30-min intervals using a Varioskan Flash reader (Thermo Scientific) (excitation, 538 nm; emission, 604 nm). Slopes were used for calculating the rate of H_2_O_2_ formation.

### Mitochondrial membrane potential

The mitochondrial membrane potential (Δ*ψ*_m_) was assessed through two different methodological approaches: (1) MitoProbe DiIC_1_(5) (50 nM, Life Technologies) by flow cytometry (FACSCalibur flow cytometer, BD Biosciences) and expressed in AU. For this purpose, cultured cells or adult brain cell suspensions were incubated with the probe for 30 min at 37 °C in PBS. Δ*ψ*_m_ was obtained after subtraction of the potential value determined in the presence of carbonyl cyanide 4-(trifluoromethoxy)phenylhydrazone (10 µM, 15 min) for each sample; (2) TMRM (10 nM, Sigma-Aldrich) with cyclosporine H (1 μM, Sigma-Aldrich) by confocal microscopy using the Operetta CLS microscope (PerkinElmer). For this aim, primary neurons were seeded into 96-well plates (PerkinElmer) and pre-incubated in KRPG buffer (145 mM NaCl, 5.7 mM Na_2_HPO_4_, 4.86 mM KCl, 0.54 mM CaCl_2_, 1.22 mM MgSO_4_, 5.5 mM d-glucose, pH 7.35). Furthermore, cells were loaded with the dye TMRM (10 nM) and cyclosporine H (1 μM) in the Operetta CLS microscope (30 min at 37 °C in a 5% CO_2_ atmosphere), and confocal images were acquired with a ×40, 1.4-NA objective (PerkinElmer). The mitochondrial uncoupler carbonyl cyanide 4-(trifluoromethoxy)phenylhydrazone (10 μM, Sigma-Aldrich) was added for 15 min as a control for mitochondrial depolarization. Finally, images were analysed using Harmony software (PerkinElmer).

### Flow cytometric analysis of apoptotic cell death

Adult brain cell suspensions from the hippocampus or the MBH were incubated with APC-conjugated annexin V and 7-aminoactinomycin D (7-AAD) (Becton Dickinson Biosciences) to quantitatively determine the percentage of apoptotic neurons (Syn-GFP^+^) by flow cytometry. Brain cell suspensions were stained with annexin V–APC and 7-AAD in binding buffer (100 mM HEPES, 140 mM NaCl, 2.5 mM CaCl_2_), according to the manufacturer’s instructions, and 10^4^ cells were analysed, in three replicates per condition, on a FACSCalibur flow cytometer (15-mW argon ion laser, CellQuest software, Becton Dickinson Biosciences), using FL4 and FL3 channels, respectively. Annexin^+^ and 7-AAD^−^ cells were considered apoptotic. The analyser threshold was adjusted on the flow cytometer channel to exclude most of the subcellular debris to reduce background noise owing to neurite disruption during neuronal resuspensions. Data were expressed as percentages.

### Determination of metabolic fluxes

To assess glycolysis, PPP and lipogenesis fluxes, we used radiometric approaches. To do this, neurons were seeded in 8-cm^2^ flasks with a hanging microcentrifuge tube containing either 1 ml benzethonium hydroxide (Sigma) (for ^14^CO_2_ equilibration) or 1 ml water (for ^3^H_2_O equilibration). All incubations were carried out in KRPG (NaCl, 145 mM; Na_2_HPO_4_, 5.7 mM; KCl, 4.86 mM; CaCl_2_, 0.54 mM; MgSO_4_, 1.22 mM; pH 7.35) containing 5 mM d-glucose at 37 °C in the air-thermostated chamber of an orbital shaker. To ensure adequate oxygen supply for oxidative metabolism throughout the incubation period, flasks’ atmosphere was gassed with carbogen (5% CO_2_ and 95% O_2_) before sealing with a rubber cap. To measure the carbon flux from glucose to CO_2_, cells were incubated in KRPG (5 mM glucose) buffer with 0.25 µCi ml^−1^ of [6-^14^C]glucose or [1-^14^C]glucose^3^. Incubations were terminated after 90 min by the addition of 0.2 ml 20% perchloric acid (Merck Millipore), and, after a further 60 min, the tube containing benzethonium hydroxide (with the trapped ^14^CO_2_) was used to determine radioactivity using a liquid scintillation analyser (Tri-Carb 4810TR, PerkinElmer). The flux of lipogenesis was measured by assaying the rate of [U-^14^C]glucose incorporation into lipids with a similar strategy using 3 μCi ml^−1^ of d-[6-^14^C]glucose in KRPG buffer (5 mM d-glucose) for 3 h^53^. After incubations were terminated with 0.2 ml 20% perchloric acid, the cells were washed twice with PBS, recollected and centrifuged at 500*g* for 5 min. The supernatant was discarded, and the pellet was resuspended in 500 μl of a chloroform–methanol mixture (2:1, vol/vol)^54^ for 16 h at −20 °C. The extract was washed with 250 μl of 0.3% (wt/vol) NaCl saturated with chloroform. The samples were centrifuged at 1,500*g* for 15 min, and the aqueous phase, containing cellular hydrosoluble components, was discarded. Later, this same process was repeated, this time using 250 μl of 0.3% (wt/vol) NaCl saturated with chloroform plus 180 μl methanol. The resulting chloroformic phase was passed to a new tube. Every step was performed at 4 °C. An aliquot of 50 μl of the organic phase containing the lipid fraction was used for the measurement of radioactivity incorporated into total lipids. Glycolytic flux was measured by assaying the rate of ^3^H_2_O production from [3-^3^H]glucose using a similar strategy with 3 μCi ml^−1^ d-[3-^3^H]glucose in KRPG buffer (5 mM d-glucose) for 120 min^3^. After incubations were terminated with 0.2 ml 20% perchloric acid, the cells were further incubated for 72 h to allow for ^3^H_2_O equilibration with water present in the central microcentrifuge tube. The ^3^H_2_O level was then measured by liquid scintillation counting (Tri-Carb 4810TR, PerkinElmer). Specific radioactivity was used for calculations. Under these experimental conditions, 75% of the produced ^14^CO_2_ and 28% of the produced ^3^H_2_O were recovered and taken into account for the calculations^3^.

### Lactate and glucose determinations

Lactate concentrations were measured in the culture medium spectrophotometrically^3^ by determination of increments in the absorbance of the samples at 340 nm in a mixture containing 1 mM NAD^+^, 8.25 U lactate dehydrogenase in 0.25 M glycine, 0.5 M hydrazine and 1 mM ethylenediaminetetraacetic acid (EDTA) buffer, pH 9.5. d-glucose was measured spectrophotometrically^3^ by reading the increase in NADPH(H^+^) absorbance at 340 nm produced in two coupled reactions catalysed by hexokinase and glucose-6-phosphate dehydrogenase (G6PD) (Roche Diagnostics) after 10 min of incubation.

### Malate–aspartate and glycerol phosphate shuttles

These were determined both by the brain concentrations of their component metabolites (aspartate, glutamate, α-ketoglutarate and malate for the malate–aspartate shuttle; glycerol 3-phosphate and dihydroxyacetone phosphate for the glycerol phosphate shuttle) and by their activities as assessed by the following ratios of the ^13^C-enriched components after [U-^13^C]glucose administration, namely: malate–aspartate and glutamate–α-ketoglutarate (malate–aspartate shuttle) and glycerol 3-phosphate–(2- and 3-phosphoglycerate) (glycerol phosphate shuttle). The sum of 2- and 3-phosphoglycerate was used as a proxy of dihydroxyacetone phosphate, which was not detected by MS.

### Oxygen-consumption rate assessment

OCRs of primary neurons were measured in real-time in an XFe24 Extracellular Flux Analyzer (Seahorse Bioscience, Seahorse Wave Desktop software 2.6.1.56). This equipment measures the extracellular medium O_2_ flux changes of cells seeded in 24-well plates. Regular cell medium was removed, and cells were washed twice with DMEM running medium (XF assay modified, supplemented with 5 mM glucose, 2 mM l-glutamine, 1 mM sodium pyruvate, 5 mM HEPES, pH 7.4) and incubated at 37 °C without CO_2_ for 30 min to allow cells to pre-equilibrate with the assay medium. Oligomycin, FCCP and a mixture of rotenone and antimycin, diluted in DMEM running medium, were loaded into port A, port B and port C, respectively. Final concentrations in XFe24 cell culture microplates were 1 μM oligomycin, 2 μM FCCP, 1 μM rotenone and 1 μM antimycin. The sequence of measurements was as follows. The basal level of the OCR was measured three times, and then reagents were injected into port A and mixed for 3 min; after, the OCR was measured three times for 3 min. The same protocol was repeated with port B and port C. OCR was measured after each injection to determine mitochondrial or non-mitochondrial contribution to the OCR. All measurements were normalized to average three measurements of the basal (starting) level of the cellular OCR of each well. Each sample was measured in three to five replicates. Experiments were repeated three times in biologically independent culture preparations. Non-mitochondrial OCR was determined by the OCR after injecting antimycin and rotenone. Maximal respiration was determined by the maximum OCR rate after FCCP injection minus the non-mitochondrial OCR. ATP production was determined by the last OCR measurement before oligomycin injection minus the minimum OCR measurement after oligomycin injection.

### Activity of mitochondrial complexes

Cells were collected and suspended in PBS (pH 7.0). After three cycles of freeze–thawing, to ensure cellular disruption, CI, complex II–III and citrate synthase activities were determined. Rotenone-sensitive NADH–ubiquinone oxidoreductase activity (CI)^55^ was measured in KH_2_PO_4_ (20 mM, pH 7.2) in the presence of 8 mM MgCl_2_, 2.5 mg ml^−1^ BSA, 0.15 mM NADH and 1 mM KCN. Changes in absorbance at 340 nm (30 °C) (*ε* = 6.81 mM^−1^ cm^−1^) were recorded after the addition of 50 µM ubiquinone and 10 µM rotenone. Deactive CI was determined after *N*-ethylmaleimide (NEM) treatment of cell homogenates (10 mM, 15 min, 15 °C). CI activity in the presence of NEM exclusively reflects the active form of CI, as NEM blocks the transition from the deactive conformation to the active conformation. Complex II–III (succinate–cytochrome c oxidoreductase) activity^56^ was determined in the presence of 100 mM phosphate buffer with 0.6 mM EDTA(K^+^), 2 mM KCN and 200 µM cytochrome c. Changes in absorbance were recorded (550 nm, 30 °C) (*ε* = 19.2 mM^−1^ cm^−1^) after the addition of 20 mM succinate and 10 µM antimycin A. Citrate synthase activity^57^ was measured in the presence of 93 mM Tris-HCl, 0.1% (vol/vol) Triton X-100, 0.2 mM acetyl-CoA, 0.2 mM 5,5′-dithiobis(2-nitrobenzoic acid) (DTNB); the reaction was started with 0.2 mM oxaloacetate, and the absorbance was recorded at 412 nm (30 °C) (*ε* = 13.6 mM^−1^ cm^−1^).

### Determination of total, reduced and oxidized glutathione

Cells were lysed with 1% (wt/vol) sulfosalicylic acid and centrifuged at 13,000*g* for 5 min at 4 °C, and the supernatants were used for determining total glutathione (GSH concentration plus twice the concentration of GSSG), by using GSSG (0–50 μM) as a standard. Total glutathione was measured in reaction buffer (0.1 mM NaHPO_4_, 1 mM EDTA, 0.3 mM DTNB, 0.4 mM NADPH and glutathione reductase at 1 U ml^−1^, pH 7.5) by recording the increase in absorbance at 405 nm after the reaction of GSH with DTNB for 2.5 min at 15-s intervals using a Varioskan Flash reader (Thermo Fisher). GSSG levels were quantified after derivatization of GSH with 2-vinylpyridine, by using similarly treated GSSG standards (0–5 μM), and results are expressed as the GSSG/GSH ratio.

### NAD^+^ determination

To determine oxidized NAD^+^ levels, we used a bioluminescent NAD/NADH-Glo Assay (Promega). Neurons were seeded in 96-well plates and incubated for 30–60 min with NAD/NADH-Glo Detection Reagent containing reductase, reductase substrate, NAD cycling enzyme and NAD cycling substrate, according to the manufacturer’s recommendations. Detergent was present in the reagent-lysed cells, allowing detection of total cellular NAD^+^. Due to the cycling of the coupled enzymatic reactions, the light signal will continue to increase after adding the reagent to the sample. The luminescent signal remains proportional to the starting amount of NAD^+^ within the linear range of the assay. To measure only the oxidized (NAD^+^) form, it is necessary to treat cells with 25 μl of 0.4 M HCl to destroy the reduced forms (NADH). Results are expressed as mean ± s.e.m. (pmol per mg protein) using the standard curve (0–400 nM).

### Sirtuin activity assay

To evaluate sirtuin activity with either HDAC or monoribosyltransferase activity, we used the fluorometric Sirtuin Activity Assay Kit (K324-100, BioVision). Neurons were seeded in 96-well plates and lysed with 300 μl of cold homogenization buffer containing protease inhibitor cocktail. Next, lysates were transferred to a cold microcentrifuge tube and agitated on a rotary shaker at 4 °C for 15 min. Finally, 50 μl cell homogenate was incubated at 37 °C for 30–60 min with 40 μl reaction mix. After incubation, 10 μl developer was added to each sample except the standards (p53–AFC substrate, 0–1,000 pmol per well), and samples were incubated for 15 min at 37 °C. Next, fluorescence was recorded (excitation and emission, 400 and 505 nm) in endpoint mode. In this protocol, the acetylated p53–AFC substrate is deacetylated by sirtuins in the presence of NAD^+^ to generate the deacetylated p53–AFC substrate, nicotinamide and *O*-acetyl-ADP-ribose. Cleavage of the deacetylated p53–AFC substrate by the developer releases the fluorescent group (AFC, 400 and 505 nm). Trichostatin A is added to the reaction to specifically inhibit HDACs in samples.

### Fructose-2,6-bisphosphate determination

For F26BP determination, cells were lysed in 0.25 M NaOH and centrifuged (20,000*g*, 20 min). An aliquot of the homogenate was used for protein determination, and the remaining sample was heated at 80 °C (5 min) and centrifuged (20,000*g*, 10 min, 4 °C), and the resulting supernatant was used for enzymatic determination of F26BP concentrations using F26BP standards, as previously described^58^. The F26BP standard was prepared by incubating a 20-mM solution of d-fructose-1,2-cyclic 6-bisphosphate (Sigma, 68872) in 0.5 M NaOH at 37 °C for 30 min. To determine F26BP levels, 20 µl of the supernatant was diluted 1:20 in 0.05 M HCl. In parallel, a non-acidified reaction was carried out by diluting the supernatant 1:10 in 0.05 M NaCl. After a 10-min incubation at room temperature, which quantitatively converts F26BP into F6P, the samples were neutralized with 0.1 M NaOH and used to determine F6P in a buffer containing 25 mM HEPES (pH 8.2), 25 mM KCl, 2.5 mM magnesium acetate, 1 mM dithiothreitol and 0.25 mM NADP^+^ in the presence of G6PD and phosphoglucose isomerase. F26BP relative levels in the samples were assessed by the coupled enzymatic activities of PFK1 (Sigma, F2258) in the presence of 1 mM F6P (Sigma, F3627) and 0.5 mM pyrophosphate (Sigma, P8010), aldolase (Sigma, A8811) and triosephosphate isomerase or glycerol-3-phosphate dehydrogenase (Sigma, G1881). This reaction generates glycerol 3-phosphate and oxidizes NADH (Sigma, N8129), producing a reduction in the absorbance at 340 nm that is monitored spectrophotometrically. F26BP concentrations are calculated in the samples by comparing the fold activation that they cause on PFK1 activity compared against an F26BP standard curve.

### Protein determination

Protein samples were quantified with the BCA Protein Assay Kit (Thermo) using BSA as a standard.

### Western blotting

Cells were lysed in RIPA buffer (1% sodium dodecyl sulphate, 10 mM EDTA, 1% (vol/vol) Triton X-100, 150 mM NaCl and 10 mM Na_2_HPO_4_, pH 7.0), supplemented with protease inhibitor mixture (Sigma), 100 μM phenylmethylsulfonyl fluoride and phosphatase inhibitors (1 mM *o*-vanadate). Samples were boiled for 5 min. Aliquots of cell lysates (40–60 μg of protein) were subjected to SDS–PAGE on an 8–15% (vol/vol) acrylamide gel (Mini-PROTEAN, Bio-Rad) including the PageRuler Prestained Protein Ladder (Thermo). The resolved proteins were transferred electrophoretically to nitrocellulose membranes (0.2 µm, Bio-Rad). Membranes were blocked with 5% (wt/vol) low-fat milk in TTBS (20 mM Tris, 150 mM NaCl and 0.1% (vol/vol) Tween-20, pH 7.5) for 1 h. After blocking, membranes were immunoblotted with primary antibodies overnight at 4 °C. After incubation with horseradish peroxidase-conjugated goat anti-mouse IgG–HRP (170-6516, Bio-Rad, 1/10,000), rabbit anti-goat IgG–HRP (sc-2768, Santa Cruz, 1/10,000) and goat anti-rabbit IgG–HRP (170-6515, Bio-Rad, 1/10,000) antibodies, membranes were immediately incubated with WesternBright ECL from the enhanced chemiluminescence kit (Advansta) or SuperSignal West Femto substrate (Thermo) before exposure to Fuji Medical X-Ray film (Fujifilm), and the autoradiograms were scanned. At least three biologically independent replicates were always performed, although only one representative western blot is shown in the main figures. The protein abundances of all western blots per condition were measured by densitometry of the bands on the films using ImageJ 1.48u4 software (National Institutes of Health) and were normalized to that of the loading control protein. The resulting values were used for statistical analysis. Uncropped scans of western blots replicates are shown in the source data.

### Primary antibodies for western blotting

Immunoblotting was performed with anti-LC3B (1/1,000) (2775, Cell Signaling), anti-p62 (1/1,000) (P0067, Sigma), anti-ATG7 (1/1,000) (2631, Cell Signaling), anti-acetylated lysine (1/1,000) (9441, Cell Signaling), anti-cyclin B1 (clone D-11, sc-7393, Santa Cruz Biotechnology, 1/500), anti-ROCK2 (clone D-11, sc-398519, Santa Cruz Biotechnology, 1/500), anti-PLIN2 (ab52356, Abcam, 1/500), anti-beclin (1/1,000) (3495, Cell Signaling), anti-TOMM20 (1/1,000) (ab56783, Abcam) anti-PFKFB3 (1/500) (H00005209-M08, Novus Biologicals), anti-VDAC (1/1,000) (PC548, Calbiochem), anti-heat-shock protein 60 (HSP60) (1/1,000) (ab46798, Abcam), anti-NDUFS1 (1/500) (sc-50132, Santa Cruz Biotechnology), anti-NDUFA9 (1/1,000) (ab14713, Abcam), anti-UQCRC2 (1/1,000) (ab14745, Abcam), anti-SDHA (1/1,000) (ab14715, Abcam), anti-ATPβ (1/1,000) (MS503, MitoSciences), anti-GFAP (1/500) (G6171, Sigma), anti-TUJ1 (1/300) (T2200, Sigma) and anti-β-actin (1/30,000) (A5441, Sigma) antibodies.

### Mitochondrial isolation

To obtain the mitochondrial fraction, cell pellets were frozen at −80 °C and homogenized (10–12 strokes) in a glass–Teflon Potter–Elvehjem homogeniser in buffer A (83 mM sucrose and 10 mM MOPS, pH 7.2). The same volume of buffer B (250 mM sucrose and 30 mM MOPS) was added to the sample, and the homogenate was centrifuged (1,000*g*, 5 min) to remove unbroken cells and nuclei. Centrifugation of the supernatant was then performed (12,000*g*, 3 min) to obtain the mitochondrial fraction, which was washed with buffer C (320 mM sucrose, 1 mM EDTA and 10 mM Tris-HCl, pH 7.4)^48^. Mitochondria were suspended in buffer D (1 M 6-aminohexanoic acid and 50 mM Bis-Tris-HCl, pH 7.0).

### Blue native gel electrophoresis

For the assessment of CI organization, digitonin-solubilized (4 g per g) mitochondria (10–50 μg) were loaded in NativePAGE Novex 3–12% (vol/vol) gels (Life Technologies). After electrophoresis, in-gel NADH dehydrogenase activity was evaluated, allowing the identification of individual CI and CI-containing supercomplex bands due to the formation of purple precipitate at the location of CI^48^. Briefly, gels were incubated in 0.1 M Tris-HCl buffer (pH 7.4), 1 mg ml^−1^ nitro blue tetrazolium and 0.14 mM NADH. Next, a direct electrotransfer was performed, followed by immunoblotting for mitochondrial CI with anti-NDUFS1 antibody (1/500) (sc-50132, Santa Cruz Biotechnology), anti-NDUFA9 antibody (1/1,000) (ab14713, Abcam), anti-complex II antibody SDHA (1/1,000) (ab14715, Abcam), anti-complex III antibody UQCRC2 (1/1,000) (ab14745, Abcam), anti-complex IV antibody MTCO4 (1/1,000) (ab14705, Abcam) and anti-complex V antibody ATPβ (1/1,000) (MS503, MitoSciences). Direct transfer of BNGE was performed after soaking the gels for 20 min (4 °C) in carbonate buffer (10 mM NaHCO_3_, 3 mM Na_2_CO_3_·10H_2_O, pH 9.5–10). Protein transfer to polyvinylidene fluoride membranes was carried out at 300 mA and 60 V for 1.5 h at 4 °C in carbonate buffer.

### Autophagic flux measurement

To analyse the autophagy pathway, primary neurons or MBH slices were incubated in the absence or the presence of inhibitors of lysosomal proteolysis, leupeptin (100 mM) and ammonium chloride (20 mM), for 2 h. Cells or tissues were lysed and immunoblotted for LC3-II, p62 and beclin 1 to assess autophagy and for TOMM20 and VDAC to assess mitophagic flux.

### Metabolomics analysis

One hemisphere from 5–6-month-old *CamkIIα-Pfkfb3* mice or wild-type mice (*n* = 6 for each condition) was snap frozen in liquid nitrogen and used for untargeted metabolomic analysis (Metabolon). Ultra-high-performance liquid chromatography–tandem mass spectroscopy analysis detected 567 metabolites. Raw data were extracted, peak identified, processed for quality control, curated and normalized by the Metabolon service. Peaks were quantified with the area under the curve. For studies spanning multiple days, a data-normalization step was performed to correct variation resulting from instrument interday tuning differences. Essentially, each compound was corrected in run day blocks by registering the medians to equal one (1.00) and normalizing each data point proportionately. Following log_2_ transformation and imputation of missing values, with the minimum observed value for each compound, Welch’s two-sample *t*-tests were used to identify compounds with significantly different concentrations between experimental groups. Metabolites labelled as xenobiotics were discarded, resulting in a total of 555 metabolites included in the final analysis. Given the high false discovery rate, metabolites were filtered according to several criteria other than the *q* value. Such criteria included (1) biological relevance given the genetic background context, (2) inclusion in a common pathway with a highly significant compound, (3) residing in a similar functional biochemical family with other significant compounds or (4) correlation with other experimental in vivo approaches, namely, magnetic resonance spectroscopy and MS. Graphs corresponding to statistical analysis were made with GraphPad Prism 8.0 software and the online tool MetaboAnalyst 5.0 (as of February 2023).

### AZ67 in vivo administration

AZ67 (Tocris) for in vivo usage was dissolved in 20% (wt/vol) PEG 200 in PBS to a concentration of 20 mM. Vehicle- and AZ67-treated *Cln7*^Δex2^ mice were used for this experiment. The cannula was inserted intracerebroventricularly at the age of 8 weeks and, after at least 15 d of recovery, we injected AZ67 at the dose identified previously (1 nmol per mouse) every 24 h^10^. The duration of the experiment was determined by the presence of hindlimb clasping in *Cln7*^Δex2^ vehicle-treated mice, this being at 2.5 months. Weight gain of the animals was monitored during this period.

### Behavioural tests

Mice (3 or 8 months old) were left to acclimatize in the room for not less than 30 min at the same time slot of the day (14:00–20:00). Tracking was carried out one at a time, and the apparatus was carefully cleaned with 70% ethanol between trials to remove any odour cues. An ANY-box core was used, which contained a light grey base and an adjustable perpendicular stick holding a camera and an infrared photo-beam array to track animal movement and to detect rearing behaviour, respectively. Mouse movements were tracked with ANY-maze software and the ANY-maze interface to register all parameters described subsequently. For the open-field test, a 40-cm × 40-cm × 35-cm (*w* × *d* × *h*) black infrared transparent Perspex insert was used, and the arena was divided into three zones, namely, border (8 cm wide), centre (16% of total arena) and intermediate (the remaining area). The test lasted for 10 min; the distance travelled and the time spent in each zone were measured.

The rotarod test (rotarod apparatus, model 47600, Ugo Basile) was used to analyse motor balance and coordination. Mice were previously trained for 3 consecutive days before testing to establish the animal’s baseline. When performing the test, the animals are placed in the cylinder under a constant acceleration of 7.2 rpm. When the test starts, the cylinder starts accelerating until it reaches 40 rpm after 5 min. The time it takes for the animal to fall from the cylinder is recorded.

The treadmill test was used to evaluate endurance (running time) and running speed (slowness). Mice were previously acclimatized for 3 consecutive days (2 h per day) to allow animals to become familiar with the treadmill and to minimize psychological stress. During this period, the electric grid remains off and the belt motor remains on but not moving. After training, animals were submitted to a graded intensity treadmill test (Model 1050 LS Exer3/6, Columbus Instruments). After a warmup period, the treadmill band velocity was increased until the animals were unable to run further. The initial bout of 6 min at 6 m × min^−1^ was followed by consecutive 2-m × min^−1^ increments every 2 min. Exhaustion was defined as the third time a mouse could no longer keep pace with the speed of the treadmill and remained on the shock grid for 2 s rather than running. Exercise motivation was provided for all rodents by means of an electronic shock grid at the treadmill rear. However, the electric shock was used sparingly during the test.

To analyse short-term memory, we used the novel object-recognition test (Stoelting) in a 40-cm × 40-cm × 35-cm (*w* × *d* × *h*) core with a black infrared transparent Perspex insert, also tracked with ANY-maze software and the ANY-maze interface to register the track of the mice. Mice were accustomed to this environment for 10 min during 2 consecutive days, and the test was performed on the third day. Mice were left to explore two identical equidistant cubes for 5 min (the familiarization phase) and returned for 15 min into their cage. One cube was substituted for a sphere of similar size and colour, and mice were returned to the arena to explore the objects for another 5 min (the test phase). To score zone entries that include the exploration of an object, we considered the size of the object (3.8 × 3.8 cm) and the surrounding perimeter (6 × 6 cm). The ability to recognize the sphere as a novel object was determined as the discrimination index (DI), calculated as DI = (*T*_N_ − *T*_F_)/(*T*_N_ + *T*_F_), where *T*_N_ is the time spent exploring the new object (sphere) and *T*_F_ is the time spent exploring the familiar object (cube).

### Magnetic resonance spectroscopy

Localized [^1^H]MRS was performed at 11.7 tesla using a 117/16 USR Bruker BioSpec system (Bruker BioSpin) interfaced to an advance III console and operating ParaVision 6.1 under TopSpin software (Bruker BioSpin). After fine-tuning and shimming of the system, water signal FWHM values typically in the 15–25-Hz range were achieved. Scanning started with the acquisition of three scout images (one coronal, one transverse and one sagittal) using a 2D-multiplane T2W RARE pulse sequence with Bruker’s default parameters. These images were used to place the spectroscopy voxel of size 1.5 × 1.5 × 2 mm^3^ located at the right striatum of the mouse brain or 2 × 0.8 × 2 mm^3^ located in the cortex (at the midline of the brain), always with care not to include the ventricles in the voxel (the geometry of the voxel was slightly altered to avoid this event, when necessary). At least two ^1^H-MRS spectra were acquired per scanning session per animal (5-month-old animals). The voxel was repositioned, and shimming adjustments were repeated between acquired spectra, when the spectral resolution of the obtained ^1^H spectrum was not good. For ^1^H-MR, a water-suppressed PRESS sequence was used with the following parameters: echo time, 17.336 ms (TE1 = TE2 = 8.668 ms); repetition time, 2,500 ms; Naverages, 256; acquisition size, 2,048 points; spectral width, 11 ppm (5,498.53 Hz). MR spectra were fitted and quantified using LCModel 6.3-1R^59^.

### Metabolite extraction for LC–MS analysis

Metabolites were extracted from plasma samples (thawed on ice) by adding 1 ml of cold methanol–water (4:1) solution to 20 µl plasma. After thorough mixing and incubation at −20 °C for 15 min, samples were centrifuged (16,000*g*, 15 min, 4 °C). The supernatant was recovered, dried using the miVac Concentrator (Genevac) and resuspended in 150 µl acetonitrile–water (4:1) solution at −20 °C with 15 mM ammonium acetate. The extract was transferred to LC–MS autosampler vials for analysis. Frozen brains were first freeze–dried and ground to powder using a Mixer Mill MM 400 (Retsch) operated with dry ice. An amount of powder corresponding to 20 mg of fresh brain tissue was then mixed with −20 °C methanol–acetonitrile–water (2:2:1) solution containing 0.1% formic acid, followed by vortexing for 1 min, incubation at −20 °C for 15 min and centrifugation (16,000*g*, 15 min, 4 °C). The supernatant was recovered, dried and resuspended in 150 µl acetonitrile–water (4:1) solution at −20 °C with 15 mM ammonium acetate. The extract was transferred to LC–MS autosampler vials for analysis.

### Liquid chromatography–mass spectrometry analysis

LC–MS analyses were performed using an UHPLC Vanquish Flex chromatographic system coupled to an Orbitrap Q Exactive+ mass spectrometer (Thermo Fisher Scientific) operated in negative (ESI−) or positive (ESI+) electrospray ionization mode. Metabolites were separated on a P120 HILIC-Z (2.1 × 150-mm i.d., 2.7 µm) column (Agilent) at 30 °C. The HILIC solvents were A, acetonitrile–water (9:1) with 15 mM ammonium acetate and B, acetonitrile–water (1:9) with 15 mM ammonium acetate. HILIC separation was performed at 250 μl min^−1^ with the following gradient (minutes, percent B): 0, 15%; 4, 25%; 5.5, 30%; 13.5, 35%; 15.9, 50%. The column was then equilibrated for 10 min at the initial conditions before the next sample was analysed. The injection volume was 2–5 μl. MS analyses were performed in full-scan mode at a resolution of 70,000 (at 400 *m*/*z*) over the *m*/*z* range 60–1,000. Data were acquired with the following source parameters: capillary temperature, 250 °C; source heater temperature, 350 °C; sheath gas flow rate, 45 AU; auxiliary gas flow rate, 10 AU; sweep gas flow rate, 1.0 AU; S-Lens RF level, 55%; source voltage, 2.70 kV (ESI− mode) or 3.50 kV (ESI+ mode). The data were acquired in a single analytical batch. Biological samples were randomized in the analytical run, and control quality (QC) samples, consisting of a mix of all biological samples of the same type (that is, plasma and brain samples, respectively), were injected at regular intervals throughout the experiment. Metabolites were identified by extracting the exact mass with a tolerance of 5 ppm. The raw MS isotopic profiles of metabolites were then quantified using TraceFinder (Thermo Fisher Scientific). Isotopologue fractions were obtained after correcting for natural isotopic abundances using IsoCor^60,61^ (https://github.com/MetaSys-LISBP/IsoCor). Molecular ^13^C enrichments were calculated from the sum of the relative abundances of all isotopologues of a metabolite weighted by the number of ^13^C atoms in each isotopologue.

### Statistical analysis

For simple comparisons, we used unpaired two-tailed Student’s *t*-test. For other multiple-value comparisons, we used one-way or two-way ANOVA followed by Tukey’s or Bonferroni post hoc tests. All tests used are indicated in each figure legend. Statistical analysis was performed using GraphPad Prism version 8 software. Numbers of biologically independent culture preparations or animals used per experiment are indicated in the figure legends. No statistical methods were used to predetermine sample sizes, but our sample sizes are similar to those reported in a previous publication^45^. Data distribution was assumed to be normal, but this was not formally tested. Data collection, assignment and organization of the experimental conditions were randomized. Data collection and analysis were not performed blind to the conditions of the experiments. No animals or data points were excluded from the analyses.

### Reporting summary

Further information on research design is available in the Nature Portfolio Reporting Summary linked to this article.

### Supplementary information

Supplementary Information Supplementary Figs. 1 and 2

Reporting Summary

Supplementary Table 1 Metabolomics data.

### Source data

Source Data Fig. 1 Source Data for Fig. 1.

Source Data Fig. 2 Source Data for Fig. 2.

Source Data Fig. 3 Source Data for Fig. 3.

Source Data Fig. 4 Source Data for Fig. 4.

Source Data Extended Data Fig. 1 Source Data for Extended Data Fig. 1.

Source Data Extended Data Fig. 2 Source Data for Extended Data Fig. 2.

Source Data Extended Data Fig. 3 Source Data for Extended Data Fig. 3.

Source Data Extended Data Fig. 4 Source Data for Extended Data Fig. 4.

Source Data Extended Data Fig. 5 Source Data for Extended Data Fig. 5.

Source Data Extended Data Fig. 6 Source Data for Extended Data Fig. 6.

Source Data Figs. 1–4 and Extended Data Figs. 1 and 3 Uncropped western blots.

Source Data Figs. 1–4 and Extended Data Figs. 1–6 Statistical table.

## Data Availability

Source data are provided with this paper.
