# Supplementary material for: Weak neuronal glycolysis sustains cognition and organismal fitness
Source: Nat Metab. 2024 May 24;6(7):1253–67. doi: 10.1038/s42255-024-01049-0 (PMC11272580; doi:10.1038/s42255-024-01049-0)
Supplement: Supplementary file 1 — Supplementary Figs. 1 and 2 [file 42255_2024_1049_MOESM1_ESM.pdf]

nature metabolism

Letter https://doi.org/10.1038/s42255-024-01049-0

Weak neuronal glycolysis sustains cognition and organismal fitness

In the format provided by the authors and unedited

Flow citometry workflow for Mitosox GFP^{+} neurons experiments

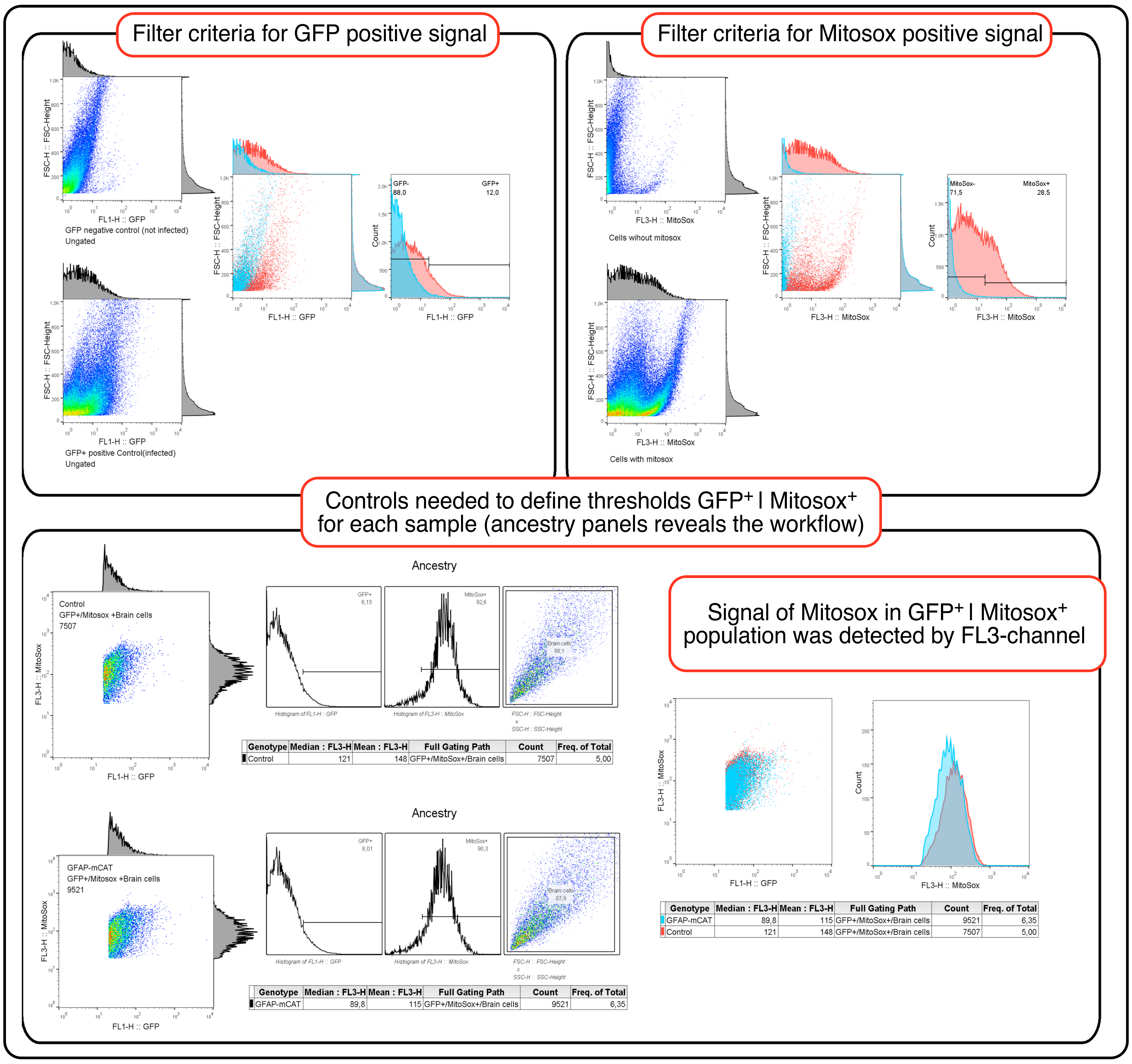

Genotype	Median : FL3-H	Mean : FL3-H	Full Gating Path	Count	Freq. of Total
Control	121	148	GFP+/MitoSox+/Brain cells	7507	5,00

Genotype	Median : FL3-H	Mean : FL3-H	Full Gating Path	Count	Freq. of Total
GFAP-mCAT	89,8	115	GFP+/MitoSox+/Brain cells	9521	6,35

Genotype	Median : FL3-H	Mean : FL3-H	Full Gating Path	Count	Freq. of Total
GFAP-mCAT	89,8	115	GFP+/MitoSox+/Brain cells	9521	6,35
Control	121	148	GFP+/MitoSox+/Brain cells	7507	5,00

Flow citometry workflow for DiIC$_1$(5) GFP^{+} neurons experiments

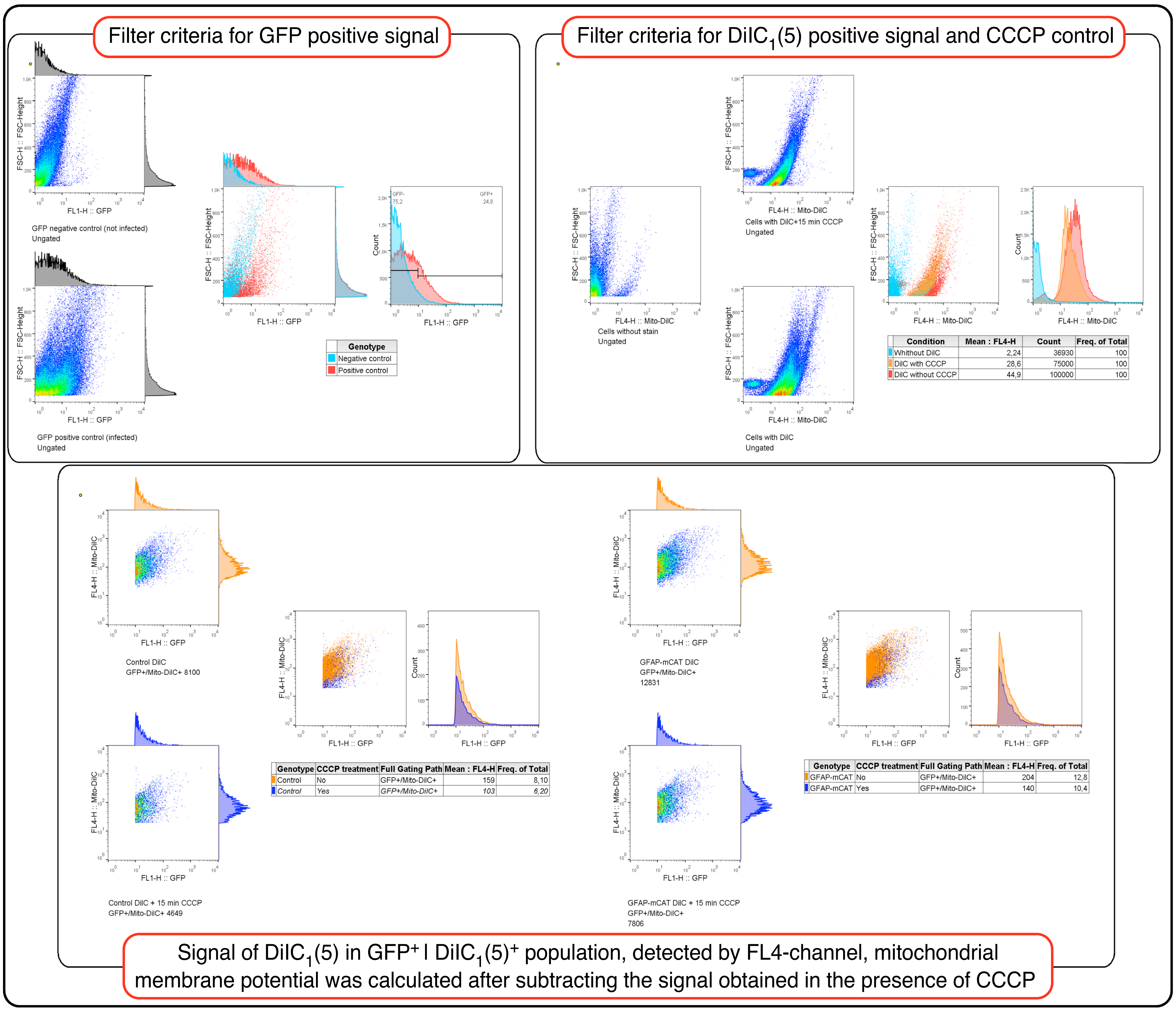

Condition	Mean : FL4-H	Count	Freq. of Total
Whithout DilC	2,24	36930	100
DilC with CCCP	28,6	75000	100
DilC without CCCP	44,9	100000	100

Genotype	CCCP treatment	Full Gating Path	Mean : FL4-H	Freq. of Total
Control	No	GFP+/Mito-DilC+	159	8,10
Control	*Yes*	*GFP+/Mito-DilC+*	*103*	*6,20*

Genotype	CCCP treatment	Full Gating Path	Mean : FL4-H	Freq. of Total
GFAP-mCAT	No	GFP+/Mito-DilC+	204	12,8
GFAP-mCAT	Yes	GFP+/Mito-DilC+	140	10,4
