# Supplementary material for: Weak neuronal glycolysis sustains cognition and organismal fitness
Source: Nat Metab. 2024 May 24;6(7):1253–67. doi: 10.1038/s42255-024-01049-0 (PMC11272580; doi:10.1038/s42255-024-01049-0)
Supplement: Supplementary file 2 — Reporting Summary [file 42255_2024_1049_MOESM2_ESM.pdf]

nature portfolio

Corresponding author(s): Juan P. Bolaños

Last updated by author(s): Apr 9, 2024

Reporting Summary

Nature Portfolio wishes to improve the reproducibility of the work that we publish. This form provides structure for consistency and transparency in reporting. For further information on Nature Portfolio policies, see our Editorial Policies and the Editorial Policy Checklist.

Statistics

For all statistical analyses, confirm that the following items are present in the figure legend, table legend, main text, or Methods section.

n/a	Confirmed	
☐	☒	The exact sample size (*n*) for each experimental group/condition, given as a discrete number and unit of measurement
☐	☒	A statement on whether measurements were taken from distinct samples or whether the same sample was measured repeatedly
☐	☒	The statistical test(s) used AND whether they are one- or two-sided<br>*Only common tests should be described solely by name; describe more complex techniques in the Methods section.*
☐	☒	A description of all covariates tested
☒	☐	A description of any assumptions or corrections, such as tests of normality and adjustment for multiple comparisons
☐	☒	A full description of the statistical parameters including central tendency (e.g. means) or other basic estimates (e.g. regression coefficient) AND variation (e.g. standard deviation) or associated estimates of uncertainty (e.g. confidence intervals)
☐	☒	For null hypothesis testing, the test statistic (e.g. *F*, *t*, *r*) with confidence intervals, effect sizes, degrees of freedom and *P* value noted<br>*Give P values as exact values whenever suitable.*
☒	☐	For Bayesian analysis, information on the choice of priors and Markov chain Monte Carlo settings
☒	☐	For hierarchical and complex designs, identification of the appropriate level for tests and full reporting of outcomes
☒	☐	Estimates of effect sizes (e.g. Cohen's *d*, Pearson's *r*), indicating how they were calculated

Our web collection on statistics for biologists contains articles on many of the points above.

Software and code

Policy information about availability of computer code

Data collection	CellQuest Pro v5, Tecnai™, QuantaSmart for TriCarb 5.1 (Perkin Elmer), AMi-maze® interface and ANY-maze® 5.33 software, Seahorse Wave Desktop Software 2.6.1.56, BioRad CFX Maestro 1.1, Harmony 4.9, Fluoview FV10-ASW, Vilber® Fusion FX6 Edge. IsoCor v2.2.2
Data analysis	Adobe Photoshop 12, ImageJ 1.48V, Paint-A-Gate ™ version PRO, GraphPad Prism v8.0, IBM SPSS 23.0, Canvas draw X, Microsoft Excel (Microsoft 365) , Fluoview FV10-ASW, Seahorse Wave Desktop Software 2.6.1.56, MetaboAnalyst 5.0.

For manuscripts utilizing custom algorithms or software that are central to the research but not yet described in published literature, software must be made available to editors and reviewers. We strongly encourage code deposition in a community repository (e.g. GitHub). See the Nature Portfolio guidelines for submitting code & software for further information.

Data

Policy information about availability of data

All manuscripts must include a data availability statement. This statement should provide the following information, where applicable:

- Accession codes, unique identifiers, or web links for publicly available datasets
- A description of any restrictions on data availability
- For clinical datasets or third party data, please ensure that the statement adheres to our policy

Source data and western blot replicas for each figure are provided with this paper in the Source Data file. Original uncropped immunoblots that support all the figures and findings of this study are also available.

Human research participants

Policy information about studies involving human research participants and Sex and Gender in Research.

Reporting on sex and gender	N/A
Population characteristics	N/A
Recruitment	N/A
Ethics oversight	N/A

Note that full information on the approval of the study protocol must also be provided in the manuscript.

Field-specific reporting

Please select the one below that is the best fit for your research. If you are not sure, read the appropriate sections before making your selection.

☒ Life sciences ☐ Behavioural & social sciences ☐ Ecological, evolutionary & environmental sciences

For a reference copy of the document with all sections, see nature.com/documents/nr-reporting-summary-flat.pdf

Life sciences study design

All studies must disclose on these points even when the disclosure is negative.

Sample size	We estimated the sample sizes for each type of experiment according to the ARRIVE guidelines in order to obtain a minimum power of 0.8 (80%) with the fewest possible animals (Krzywinski & Altman, Power and sample sizes, Nat Methods 2013, 10:1139, doi.org/10.1038/nmeth.2738; Button et al., Power failure: why small sample size undermines the reliability of neuroscience, Nat Rev Neurosci 2013, 14:365, doi.org/10.1038/nrn3475). We optimized the experimental protocols by performing pilot experiments in order to maximize the differences between conditions. In addition, we estimated the sample sizes by the previous experience of the group in in vitro molecular experiments and in vivo experiments and/or on previously published similiar experiments.<br>Exact information on the sample numbers being analyzed can be found in Figure legends and in Supplementary Information.<br>The majority of biochemical assays were repeated at least three times in order to derive statistical information such as error bars, p values and significance.
Data exclusions	No data were excluded from analyses.
Replication	In vitro: experiments were done from 3 to 5 times with independent biological samples and the necessary technical replicates for each technique (tipically, 4-6 replicas), in order to reproduce the results found. In vivo: the sample size of the behavioral studies was higher than in the in vitro experiments given the variability in the parameters measured in order to confirm a reliable result. This information has been added in the Statistical section of the manuscript. All attempts at replication were successfull. In vivo experiments were performed in 3-12 mice (usually 7-12) per condition.
Randomization	For all mouse experiments, animals were chosen based on genotypes. Aged-matched wild-type and mutant littermates were compared to minimize variance in age, genetic background and environment. Then a general method of randomization to assign experimental groups was not performed because all experiments were conducted with appropiate positive and negative controls, therefore it was not applicable.<br>For in vitro studies, randomization is not applicable as cells with different treatments or genetic knockdown cannot be randomized.
Blinding	Blinding was not considered to be necessary in biochemical, blotting and imaging experiments because they were analyzed in exactly the same manner.<br>For in vivo analysis, all experiments were done by experienced researchers blind for the experimental conditions.

Reporting for specific materials, systems and methods

We require information from authors about some types of materials, experimental systems and methods used in many studies. Here, indicate whether each material, system or method listed is relevant to your study. If you are not sure if a list item applies to your research, read the appropriate section before selecting a response.

Materials & experimental systems

n/a	Involved in the study
☐	☒ Antibodies
☒	☐ Eukaryotic cell lines
☒	☐ Palaeontology and archaeology
☐	☒ Animals and other organisms
☒	☐ Clinical data
☒	☐ Dual use research of concern

Methods

n/a	Involved in the study
☒	☐ ChIP-seq
☐	☒ Flow cytometry
☐	☒ MRI-based neuroimaging

Antibodies

Antibodies used

Primary antibodies:

GFAP (clone 2e8, G6171; Sigma ,1/500), B-Tubulin III (T2200; Sigma ,1/500), NeuN (clone A-60, MAB377; Merck, 1/1000), Cyclin B1 (clone D-11, sc-7393; Santa Cruz Biotechnology, 1/500), Rock2 (clone D-11, sc-398519; Santa Cruz Biotechnology, 1/500), Plin2 (ab52356; Abcam, 1/500), TOMM20 (clone 4F3, ab56783; Abcam, 1/1000), VDAC (clone Ab-5, PC548; Merck, 1/1000), NDUFS1 (clone E-20, sc-50132; Santa Cruz Biotechnology 1/500), UQCRC2 (clone 13G12AF12BB11, ab14745; Abcam, 1/1000), NDUFA9 (clone 20C11B11B11, ab14713; Abcam, 1/1000), SDHA (clone 2E3GC12FB2AE2, ab14715; Abcam, 1/1000), MTCO1 (clone 1D6E1A8, ab14705; Abcam, 1/1000), ATPbeta (clone 3D5, ab14730; Abcam, 1/1000), LC3B (#2775; Cell Signaling, 1/1000), p62/SQSTM1 (P0067; Sigma, 1/1000), Atg7 (#2631; Cell Signaling, 1/1000), Acetylated-Lysine (#9441; Cell Signaling, 1/1000), Beclin-1 (clone D40C5, #3495; Cell Signaling, 1/1000), PFKFB3 (clone 3F3, H00005209-M08; Novus Biologicals, 1/500), B-Actin (clone AC-15, A5441; Sigma ,1/30000), -ATPß (MS503, MitoSciences)

Secondary antibodies:

Goat anti-mouse IgG-HRP (170-6516; Bio-Rad, 1/10000), rabbit anti-goat IgG-HRP (sc-2768, Santa Cruz, 1/10000), goat anti-rabbit IgG-HRP (170-6515; Bio-Rad, 1/10000), goat anti-rabbit-Cy2 (111-225-144, Jackson Immunoresearch, 1/500), goat anti-mouse-Cy5 (115-175-003, Jackson Immunoresearch, 1/500).

Validation

Antibodies used in this study were validated by the manufacturer who provided references on their websites using the catalog number provided and/or proven to work in the following papers (references belong):

-GFAP (G6171; Sigma, 1/500) was already employed in Vinukonda G et al 2012; Tse KH et al 2014; among others.
-beta-Tubulin III (ab18207; Abcam 1/500) was already employed in Choi YS et al 2020; Navneet S et al 2019; among others.
-NeuN (clone A-60, MAB377; Merck, 1/1000) was already employed in Linden, JR et al 2015; Herculano-Houzel, S et al 2015; Zuckermann, M et al 2015; among others.
-Cyclin B1 (clone D-11, sc-7393; Santa Cruz Biotechnology, 1/500) was employed in DiFederico, E., et al. 1999; Kumar, V., et al. 2017; Fu, S., et al. 2018; among others.
-Rock2 (clone D-11, sc-398519; Santa Cruz Biotechnology, 1/500) was employed in Park, Y.H., et al. 2016; Sugimoto, W., et al. 2018; Hou, C., et al. 2018; among others.
-TOMM20 (ab56783; Abcam, 1/1000) was already employed in Darna M et al. 2009; Yarham JW et al. 2014; Zhang Y et al. 2020; Mu Y et al.2021; among others.
-VDAC (clone Ab-5, PC548; Merck, 1/1000) was already employed in Crompton, M., et al. 1999; Yu, W.H., et al. 1995; McEnry, M.W., et al. 1993; among others.
-NDUFS1 (sc-50132; Santa Cruz Biotechnology 1/500) was already employed in Martin MA et al. 2005; Duncan AM et al. 1992; among others.
- UQCRC2 (ab14745; Abcam, 1/1000) was already employed in Kremer LS et al.2017; Zhao QY et al 2020; Chen C et al.2021; among others.
-NDUFA9 (ab14713; Abcam, 1/1000) was already employed in Calvo E et al. 2020; González-García P et al. 2020; among others.
-SDHA (ab14715; Abcam, 1/1000) was already employed in Benegiamo G et al. 2022; Greggio C el al. 2017; among others.
-MTCO1 (ab14705; Abcam, 1/1000) was already employed in Balsa E et al. 2019; Greggio C el al. 2017; among others.
-ATPbeta (clone 3D5, ab14730; Abcam, 1/1000) was employed in Pinson MR et al. 2022; Li X et al. 2022; Long M et al. 2022; among others.
-LC3B (#2775; Cell Signaling, 1/1000) was employed in Reggiori, F. and Klionsky, D.J. 2002; Codogno, P. and Meijer, A.J. 2005; Ichimura, Y. et al. 2000; among others.
-p62/SQSTM1 (P0067; Sigma, 1/1000) was employed in Bjørkøy, G et al. 2005; Ralston, SH et al. 2008; Klionsky, DJ, and Emr, SD. 2000; among others.
-Atg7 (#2631; Cell Signaling, 1/1000) was employed in Reggiori, F and Klionsky, DJ. 2002; Suzuki, K et al. 2001; Mizushima, N et al. 1998; among others.
-Acetylated-Lysine (#9441; Cell Signaling, 1/1000) was employed in Boyes, J et al. 1998; Choudhary, C et al. 2009; among others.
-Beclin-1 (clone D40C5, #3495; Cell Signaling, 1/1000) was employed in Liang, XH et al. 1999; Liang, XH et al. 2001; Yue, Z et al. 2003; among others.
-PFKFB3 (clone 3F3, H00005209-M08; Novus Biologicals, 1/500) was employed in Lopez-Fabuel I and Garcia-Macia M. 2022; Burmistrova O et al. 2019; Almeida A, Bolanos JP and Moncada, 2010; among others.
- B-Actin (A5441; Sigma 1/30000) was already employed in Melanie Si Yan Tan et al. 2019; Lorraine Springuel et al 2014; among others.
- Plin2 (ab52356; Abcam, 1/500) was employed in Becker, L et al. 2010; Rongbo, L et al. 2022; among others.
-HSP60 (ab46798; Abcam) was employed in Lopez-Fabuel I et al Nat Commun 2022, among others.
-ATPß (MS503, MitoSciences) was employed by Long et al, EMBO J (2022) among others.
-Goat anti-mouse IgG-HRP (170-6516; Bio-Rad) was used by Basu S et al. 2014; Hainer SJ et al, 2016; Yamamoto S et al. 2021

-Rabbit anti-goat IgG-HRP (sc-2768, Santa Cruz) was employed by Zheng FQ et al. 2009; Bruschetta G et al. 2018
-Goat anti-rabbit IgG-HRP (170-6515; Bio-Rad) was used by Bhat UG et al. 2015; Zhu H et al. 2022
-Goat anti-rabbit-Cy2 (111-225-144, Jackson Immunoresearch) was used in Baumgartner P et al. 2018; Cerina M et al. 2020
-Goat anti-mouse-Cy5 (115-175-003, Jackson Immunoresearch) was used in Mazo C et al. 2022; Yan B et al. 2022

Animals and other research organisms

Policy information about studies involving animals; ARRIVE guidelines recommended for reporting animal research, and Sex and Gender in Research

Laboratory animals	Cdh1(lox/lox) mice (PMID: 18552834), mCat(lox/+) mice (PMID: 32694785), Pfkfb3(lox/+) mice and Cln(Δex2) mice (PMID: 35087090) were used in C57BL6/J background at the age of 2,5 to 8 months To inactivate Cdh1 gene in neurons of the adult brain, Cdh1(lox/lox) mice were mated with mice carrying the gene encoding Cre recombinase under the control of the CamkIIa promoter (The Jackson Laboratory), generating CamkIIa-Cdh1(-/-) mice.<br>Pfkfb3(lox/+) mice were generated by homologous recombination in the Rosa26 locus of embryonic stem cells under a C57BL/6 background, where we introduced the full-length cDNA of Pfkfb3 preceded by a transcriptional STOP cassette flanked by two loxP sites. This loxP-flanked STOP signal was incorporated between the CAG promoter and the Pfkfb3 cDNA. To express Pfkfb3 in neurons in vivo, Pfkfb3(lox/+) mice were mated with CamkIIa-Cre mice to generate CamkIIa-Pfkfb3.<br>mCat(lox/+) mice were mated with Pfkfb3(lox/+) mice, which generated the double transgenic Pfkfb3(lox/+);mCat(lox/+) mice. Then, Pfkfb3(lox/+);mCat(lox/+) mice were mated with CamkIIa-Cre to generate CamkIIa-Pfkfb3-mCat mice.
Wild animals	No wild animals were used in the study
Reporting on sex	Males and females were used for behavioral analysis observing the same phenotype in both sexes without any evidence of sexual dimorphism. From this moment we used only males for the rest of the study to minimize the number of animals as much as possible.
Field-collected samples	No field collected samples were used in the study.
Ethics oversight	Procedures were approved by the Bioethics Committee of the University of Salamanca or CIC bioGUNE (PET and MRS) in accordance with the Spanish legislation (RD53/2013).

Note that full information on the approval of the study protocol must also be provided in the manuscript.

Flow Cytometry

Plots

Confirm that:

☒ The axis labels state the marker and fluorochrome used (e.g. CD4-FITC).

☒ The axis scales are clearly visible. Include numbers along axes only for bottom left plot of group (a 'group' is an analysis of identical markers).

☒ All plots are contour plots with outliers or pseudocolor plots.

☒ A numerical value for number of cells or percentage (with statistics) is provided.

Methodology

Sample preparation	Primary neurons or adult brain-cell suspensions were used. These cells were treated with the corresponding probes (Mitosox, DilC1, Annexin V or 7AAD), following manufacturer instructions, during a determined time. After incubation with the corresponding probe, the cells were centrifuged and the pellets were resuspended in PBS for further analysis.
Instrument	FACScalibur flow cytometer (BD Biosciences), equipped with a 15 mW argon laser
Software	CellQuest ™ v5 for adquisition and Paint-A-Gate ™ PRO (BD Biosciences) and FlowJo v10 for data quantification.
Cell population abundance	At least 100,000 events were acquired in triplicate and by condition.
Gating strategy	The threshold of the analyzer was adjusted in the corresponding channel of the flow cytometer to exclude most subcellular residues or cellular aggregates in the SSC/FSC plot. The median intensity values were obtained for each sample, and the FMO (unstained cells) substracted.

☒ Tick this box to confirm that a figure exemplifying the gating strategy is provided in the Supplementary Information.

Magnetic resonance imaging

Experimental design

Design type	In vivo magnetic resonance spectroscopy was performed for the determination of metabolites in different brain regions.

Design specifications	Animals are kept anesthetized during measurements using 1-1.5% isoflurane. The animal's temperature is maintained around 37.0 ºC by means of a hot water circuit and its respiration and temperature are monitored during the acquisition of the MRI experiments using the SAII M1030 system (SA instruments, NY, USA).<br>The MRI session begins with the acquisition of a localization scan to continue with the established protocol, which consists of acquiring axial and sagittal anatomical images that allow selecting the final position of the voxel for spectroscopy.
Behavioral performance measures	*State number and/or type of variables recorded (e.g. correct button press, response time) and what statistics were used to establish that the subjects were performing the task as expected (e.g. mean, range, and/or standard deviation across subjects).*

Acquisition

Imaging type(s)	Structural
Field strength	All MRI experiments have been performed at 11.7 Tesla in a Bruker Biospec 117/16 with high performance gradients of 9 cm internal diameter. The experiments have been carried out using a volumetric antenna of 82 mm diameter and a surface antenna optimized for mouse brain as receiver.
Sequence & imaging parameters	After fine tuning and shimming of the system water signal FWHM values typically in the 15-25 Hz range were achieved. Scanning started with the acquisition of three scout images (one coronal, one transverse and one sagittal) using a 2D-multiplane T2W RARE pulse sequence with Bruker's default parameters. For 1H-MR a water suppressed PRESS sequence was used with the following parameters: Echo time = 17.336 ms (TE1 = TE2 = 8.668 ms); Repetition time = 2500 ms; Naverages = 256; Acquisition size = 2048 points; spectral width = 11 ppm (5498.53 Hz).
Area of acquisition	Those images were used to place the spectroscopy voxel of size 1.8 x 1.8 x 1.8 mm3 located at the right striatum of the mouse brain or 2 x 0.8 x 2 mm3 located in cortex (at the mid-line of the brain), always with care not to include the ventricles in the voxel (the geometry of the voxel was slightly altered to avoid this event, when necessary).<br>The following parameters are used: STEAM sequence, with VAPOR (Variable Pulse Power and Optimized Relaxation delays) water suppression, TE 3.7 ms, TR 4 seconds, mixing time 10 ms, number of averages 512, BW = 11 ppm, 2K complex points, ACQ time= 34m8s0ms.

Diffusion MRI ☐ Used ☐ Not used

Preprocessing

Preprocessing software	117/16 USR Bruker Biospec system (Bruker Biospin GmbH, Ettlinglen, Germany) interfaced to an advance III console and operating ParaVicion 6.1 under topspin software (Bruker Biospin). MR spectra were fitted and quantified using LC-Model 6.3-1R.
Normalization	The concentrations of the metabolites have been estimated using LCMODEL ver 6.3 (www.lcmodel.com) using a simulated base using FID-A (DOI: 10.1002/mrm.26091). Results are presented normalized to Total Creatine (PCr + CR).
Normalization template	*Describe the template used for normalization/transformation, specifying subject space or group standardized space (e.g. original Talairach, MNI305, ICBM152) OR indicate that the data were not normalized.*
Noise and artifact removal	At least two 1H-MRS spectra were acquired per scanning session per animal (5 months-old animals). The voxel was repositioned, and shimming adjustments were repeated between acquired spectra, when the spectral resolution of the obtained 1H-spectrum was not good.
Volume censoring	*Define your software and/or method and criteria for volume censoring, and state the extent of such censoring.*

Statistical modeling & inference

Model type and settings	*Specify type (mass univariate, multivariate, RSA, predictive, etc.) and describe essential details of the model at the first and second levels (e.g. fixed, random or mixed effects; drift or auto-correlation).*
Effect(s) tested	We have observed significant differences between WT and CamkIIa-Pfkfb3 mice in the concentration of some metabolites such as lactate, GSH, NAA. We have used a two-tailed-unpaired t-Test to compare both genotypes.

Specify type of analysis: ☒ Whole brain ☐ ROI-based ☐ Both

Statistic type for inference<br>(See Eklund et al. 2016)	*Specify voxel-wise or cluster-wise and report all relevant parameters for cluster-wise methods.*
Correction	*Describe the type of correction and how it is obtained for multiple comparisons (e.g. FWE, FDR, permutation or Monte Carlo).*

Models & analysis

n/a	Involved in the study
☐	☐ Functional and/or effective connectivity
☐	☒ Graph analysis
☐	☐ Multivariate modeling or predictive analysis

Functional and/or effective connectivity	*Report the measures of dependence used and the model details (e.g. Pearson correlation, partial correlation, mutual information).*
Graph analysis	*Report the dependent variable and connectivity measure, specifying weighted graph or binarized graph, subject- or group-level, and the global and/or node summaries used (e.g. clustering coefficient, efficiency, etc.).*
Multivariate modeling and predictive analysis	*Specify independent variables, features extraction and dimension reduction, model, training and evaluation metrics.*
