# Supplementary material for: Weak neuronal glycolysis sustains cognition and organismal fitness
Source: Nat Metab. 2024 May 24;6(7):1253–67. doi: 10.1038/s42255-024-01049-0 (PMC11272580; doi:10.1038/s42255-024-01049-0)

Uncropped Western Blots and their Replicas

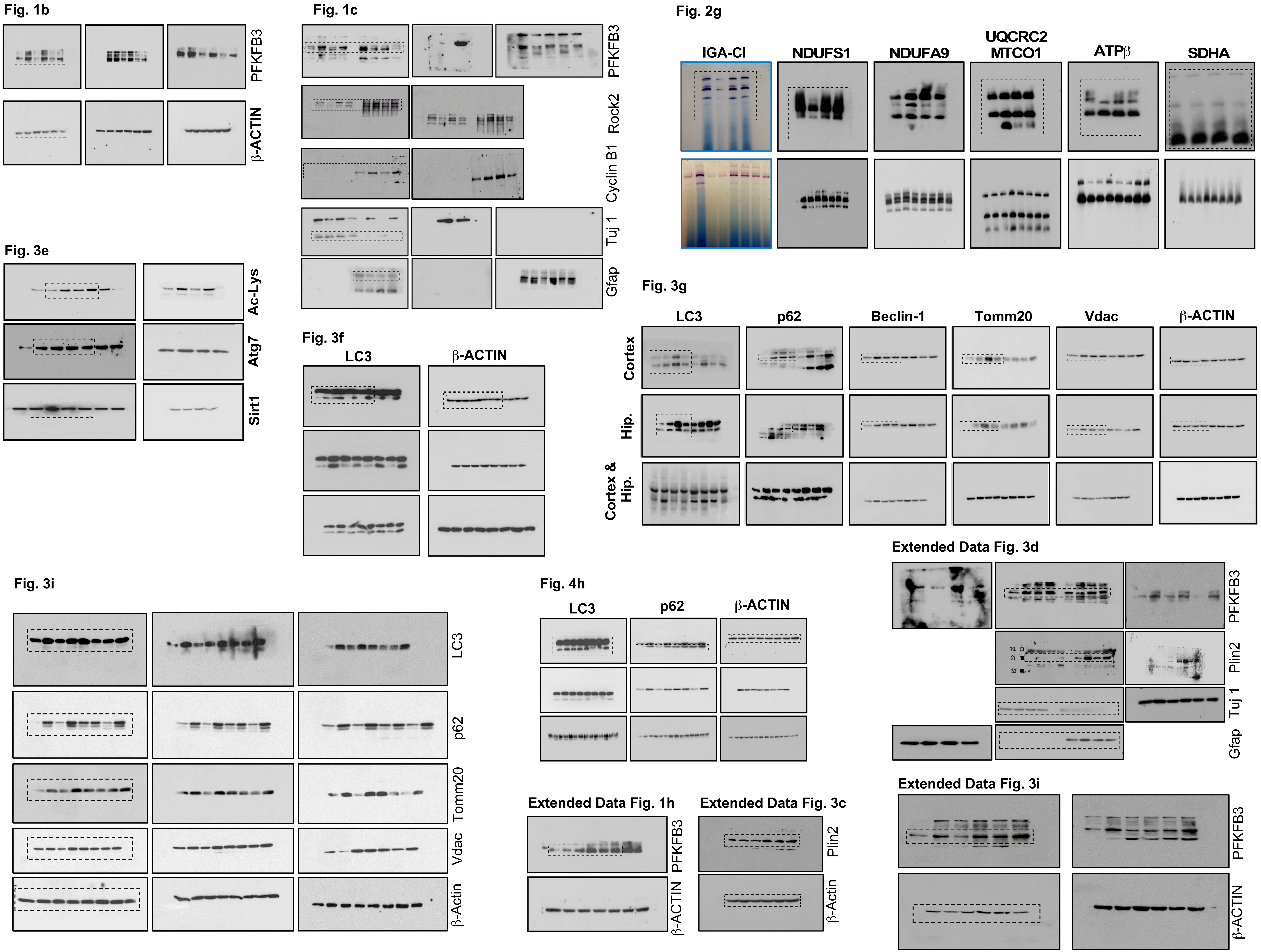

Supplement: Supplementary file 14 — Uncropped western blots. [file 42255_2024_1049_MOESM14_ESM.pdf]
